# Outer membrane lipoprotein RlpA is a novel periplasmic interaction partner of the cell division protein FtsK in *Escherichia coli*

**DOI:** 10.1038/s41598-018-30979-5

**Published:** 2018-08-28

**Authors:** Alison M. Berezuk, Sabrina Glavota, Elyse J. Roach, Mara C. Goodyear, Jonathan R. Krieger, Cezar M. Khursigara

**Affiliations:** 1 Department of Molecular and Cellular Biology, University of Guelph, Guelph, ON N1G 2W1 Canada; 2 SPARC BioCentre, The Hospital for Sick Children, Toronto, ON M5G 0A4 Canada

## Abstract

In *Escherichia coli*, formation of new cells is mediated by the elongasome and divisome that govern cell elongation and septation, respectively. Proper transition between these events is essential to ensure viable progeny are produced; however, the components of each complex responsible for transmission of the cell signal to shift from elongation to septation are unclear. Recently, a region within the N-terminal domain of the essential divisome protein FtsK (FtsK_N_) was identified that points to a key role for FtsK as a checkpoint of cell envelope remodeling during division. Here, we used site-specific *in vivo* UV cross-linking to probe the periplasmic loops of FtsK_N_ for protein interaction partners critical for FtsK_N_ function. Mass spectrometry analysis of five unique FtsK_N_ periplasmic cross-links revealed a network of potential FtsK_N_ interactors, one of which included the septal peptidoglycan binding protein rare lipoprotein A (RlpA). This protein was further verified as a novel interaction partner of FtsK_N_ by an *in vitro* pull-down assay. Deletion of *rlpA* from an FtsK temperature-sensitive *E*. *coli* strain partially restored cell growth and largely suppressed cellular filamentation compared to the wild-type strain. This suggests that interaction with RlpA may be critical in suppressing septation until proper assembly of the divisome.

## Introduction

In Gram-negative, rod-shaped bacteria, the interplay between cell growth and division is highly coordinated. As with most Gram-negative bacteria, the cell envelope of *Escherichia coli* contains three layers; a thin peptidoglycan layer enclosed within a periplasmic space by two structurally distinct cell membranes^1^. Together, these layers form an essential, selectively permeable barrier to the external environment. Based on the indispensable nature of the cell envelope, the precise, simultaneous modification and rearrangement of all three layers during growth and division is necessary to ensure strict maintenance of its barrier function at all stages.

Through the combined action of two large macromolecular protein complexes, cells undergo two broad morphological changes during division. In *E*. *coli*, lateral insertion of peptidoglycan along the long axis of the cell is facilitated by the elongasome^2,3^. Following sufficient elongation, invagination of the cell envelope is driven by the divisome complex^4,5^. Collectively, the elongasome and divisome are made up of over 30 essential and non-essential proteins^2,6^. Each complex is composed of a membrane-anchored protein scaffold and associated peptidoglycan synthesis enzymes. During elongation, positioning of the elongasome peptidoglycan synthesis machinery is driven by MreB, a homolog of the eukaryotic protein actin^7–10^. Similarly, the septal peptidoglycan synthesis machinery is anchored at mid-cell by the Z-ring, which is mainly composed of the eukaryotic tubulin homolog FtsZ^10–13^.

Although many parallels exist between the functions and general organization of each complex^2,3^, it is still unclear how the elongasome and divisome interact to integrate essential information regarding the growth state of the cell. In particular, it is unknown how the switch from dispersed lateral peptidoglycan synthesis to concentrated synthesis at the new cell poles and invagination of the cell envelope during septation occurs. The prevailing theory is that transduction of a cell signal from the peptidoglycan synthesis machinery of the elongasome to the divisome in the periplasm is then transmitted by at least one transmembrane protein to the Z-ring in the cytoplasm to initiate constriction^4,14–18^. Co-localization and protein interaction analysis of several elongasome and divisome proteins indicates that these complexes are simultaneously present at mid-cell for approximately 40% of the cell division cycle, and that the peptidoglycan synthesis enzymes from each complex physically interact prior to visible cell constriction^19^. This organization would permit transduction of the signal between the peptidoglycan synthesis proteins in the periplasm (namely penicillin binding protein 2 [PBP2] of the elongasome and FtsI [PBP3] of the divisome), but would not account for constriction initiation by the Z-ring. In the absence of proper coordination, cells lose the ability to effectively replicate their cellular material, resulting in the inability to produce viable progeny. As such, elucidating the protein-protein interactions formed between these essential complexes is critical to understand how cells transmit information during division and ensure survival.

An intriguing candidate to mediate the transmission of this critical cell signal is the essential cell division protein FtsK. FtsK is a large, multi-spanning membrane protein that is composed of 1329 amino acids and has a molecular weight of approximately 147 kDa^20,21^. It belongs to the large, well-conserved SpoIIIE family of proteins that facilitates double-stranded DNA (dsDNA) translocation *via* its C-terminal domain during division and sporulation in *E*. *coli* and *Bacillus subtilis*, respectively^22,23^. In *E*. *coli*, the essential N-terminal, membrane anchor domain of FtsK (FtsK_N_) is involved in septation^21,24,25^, although the precise role of this domain is unclear.

Based on the bifunctional nature of FtsK, it has been suggested that it may serve as a critical checkpoint of cell division in *E*. *coli*^26–30^. However, until recently, this checkpoint function was thought to merely signal completion of chromosome segregation prior to septation. With recent evidence from our laboratory regarding a novel functional periplasmic loop of FtsK_N_, we proposed a revised role for FtsK_N_ during division that involves the regulation of cell envelope remodeling events^25^. The uncoupling of cytoplasmic constriction from peptidoglycan and outer membrane invagination caused by mutation of periplasmic residues of FtsK_N_ suggests that FtsK_N_ might link the Z-ring to the periplasmic peptidoglycan synthesis machinery. While sufficient biochemical evidence suggests that FtsK_N_ interacts with the Z-ring during division^27,31–33^, interaction between FtsK and proteins involved in peptidoglycan synthesis (e.g., FtsI) has not been unequivocally verified^27,31,32^.

In the following study, the periplasmic loops of FtsK_N_ were probed by *in vivo* site-specific incorporation of an unnatural photoactivatable cross-linking residue to identify novel protein interaction partners of FtsK that may contribute to its proposed checkpoint function. Interestingly, an outer membrane lipoprotein of unknown function in *E*. *coli*, rare lipoprotein A (RlpA), was identified in all cross-linked samples within the functional periplasmic loop of FtsK_N_ (i.e., residues D135, D136 and Y139). RlpA is one of four SPOR-domain containing proteins in *E*. *coli* (including FtsN, DedA and DamX) that bind peptidoglycan and are targeted to the septum during division^34–36^. Recently, RlpA was shown to function as a lytic transglycosylase in *Pseudomonas* *aeruginosa*^37^. Deletion of *rlpA* in *P*. *aeruginosa* caused slow growth and chaining of cells when grown in a low osmotic strength medium, suggesting a role for RlpA in cell-cell separation and rod shape maintenance^37^. Despite considerable effort by several groups to determine the function of RlpA in *E*. *coli*, null mutants of *rlpA* in this species have yielded no morphological defects, nor has purified *E*. *coli* RlpA shown any enzymatic activity towards peptidoglycan^34,35,37^. Here, we show that *E*. *coli* RlpA directly interacts with divisome protein FtsK_N_ *in vitro*, and that deletion of *rlpA* partially bypasses the requirement for functional FtsK, as seen by growth and morphological analysis of an *rlpA* knockout strain.

## Results

### *In vivo* UV cross-linking approach

To identify FtsK_N_ periplasmic interaction partners, a site-specific *in vivo* UV cross-linking approach was used. This technique has been successfully used in *E*. *coli* to probe various protein interaction surfaces, including mapping of the SecA dimer interface and its interaction with the Sec translocon^38,39^, transmembrane translocation by the Tat-pathway^40^, capsular polysaccharide export by Wza^41^ and formation of the essential divisome sub-complex FtsQ/B/L^42^. An exogenous photoactivatable amino acid, *p*-benzoyl-l-phenylalanine (*p*Bpa), is incorporated into the amino acid sequence of FtsK_N_ by a mutant aminoacyl-tRNA synthetase/tRNA pair derived from *Methanococcus jannaschii*^43,44^. This suppresses an amber codon (TAG) engineered within the sequence of *ftsK*_*N*_ by incorporating the *p*Bpa residue and generates a photo-modified protein variant (FtsK_N_*). When cells expressing the photo-modified variants are exposed to long wavelength UV light, a site-specific covalent link between FtsK_N_* and any interacting protein within 3.1 Å is formed^45^. Given that residues in the second periplasmic loop of FtsK_N_ were previously found to be critical for function^25^, two functionally important residues (D135 and D136) were chosen for substitution with *p*Bpa. Further, three additional residues (W51, Y139 and L158) were also chosen for substitution (Supplementary Fig. S1). Each position was selected to spread coverage over the periplasmic loops of FtsK_N_ and verify the presence of additional protein binding sites at locations distinct from the functional region identified previously^25^. Large/aromatic amino acids were preferentially chosen for replacement to minimize disruption of protein contacts by incorporation of the bulky *p*Bpa residue.

**Figure 1:**
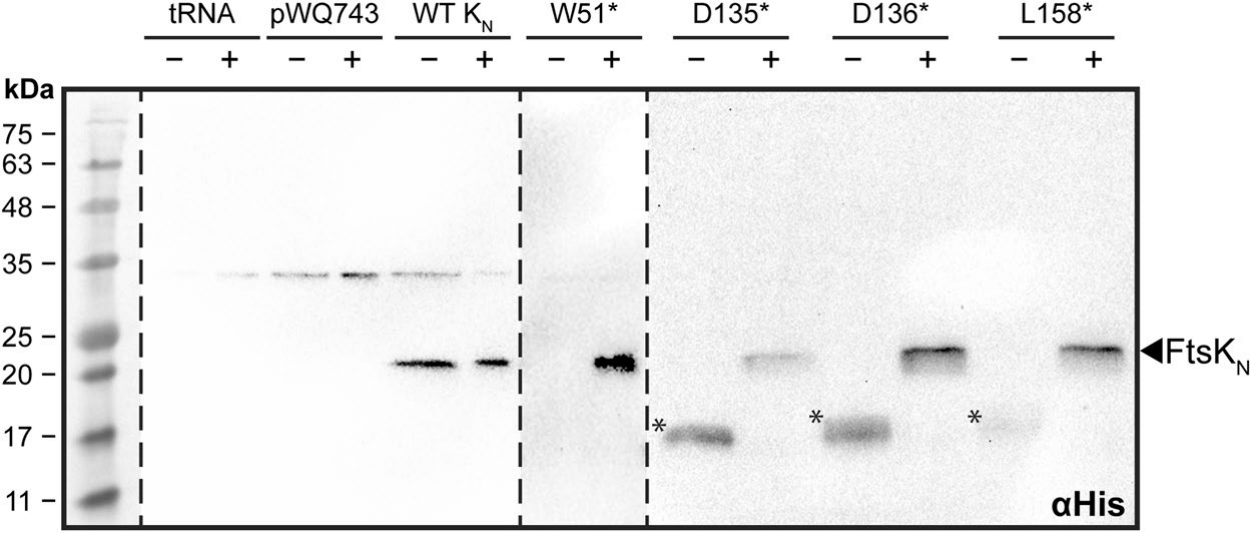
Growth of FtsK_N_ amber mutants (FtsK_N_*) in the presence of *p*Bpa restores expression of full-length FtsK_N_. Western blot analysis of FtsK_N_ amber mutant expression. Temperature-sensitive *E*. *coli* strain LP11-1 (*ftsK44*) carrying wild-type (WT) FtsK_N_ or each amber mutant was cultured at 42 °C for 2 h with (+) or without (−) 1 mM *p*Bpa. Concentrated whole cell lysates were separated by SDS-PAGE, blotted onto a nitrocellulose membrane, and subsequently probed with mouse anti-His_6_ antibodies, followed by horseradish peroxidase-conjugated goat anti-mouse IgG. tRNA and pWQ743 represent control cells harbouring the mutant tRNA/tRNA synthetase and empty vector, respectively. Asterisks (*) indicate the presence of truncated FtsK_N_ in cultures without *p*Bpa. Samples detected on separate blots are delineated by the dotted lines, and all were developed using the parameters outlined in the ‘Methods’ section. The representative molecular weight ladder was captured by taking a reference image of the undeveloped blots.

The goal of our cross-linking strategy was to capture endogenous interacting proteins. It was therefore critical to achieve near native expression levels of each FtsK_N_* variant. Using a highly tunable anhydrotetracycline (ATc)-inducible expression system^41^, expression of each FtsK_N_* variant in the presence and absence of *p*Bpa was assessed by SDS-PAGE and Western blotting. This also verified production of full-length FtsK_N_. Full-length FtsK_N_ could be detected in all samples only when *p*Bpa was added to the culture medium (Fig. 1). In the absence of *p*Bpa, truncated versions of FtsK_N_ could be detected (with the exception of W51*), highlighting the specificity of the mutant tRNA/tRNA synthetase pair and proper incorporation of the amber stop codon into the sequence of *ftsK*_*N*_. All FtsK_N_ constructs were only detectable in long exposures of the Western blot (5 min), indicating very low levels of expression were achieved.

Initially, we attempted to identify FtsK_N_ periplasmic interaction partners by affinity-purifying FtsK_N_ cross-linked products, separating the adducts by SDS-PAGE and identifying the resulting protein complex by mass spectrometry following in-gel digestion of the appropriate protein (as verified by Western blotting). However, given the low-level expression of the FtsK_N_* variants and limited abundance (50 to 300 copies per cell) of most endogenous cell division proteins^4,46,47^, repeated attempts using this process resulted in unreliable identification of adducts containing FtsK_N_. Therefore, we decided to use a more sensitive approach consisting of in-solution digestion of purified FtsK_N_ cross-linked products followed by liquid chromatography tandem-mass spectrometry (LC-MS/MS) identification of the total FtsK_N_ periplasmic interactome. This approach allowed us to identify a diverse interaction network of potential FtsK_N_ interaction partners, as described below.

### *In vivo* UV cross-linking reveals network of FtsK_N_ periplasmic interaction partners

**Figure 2:**
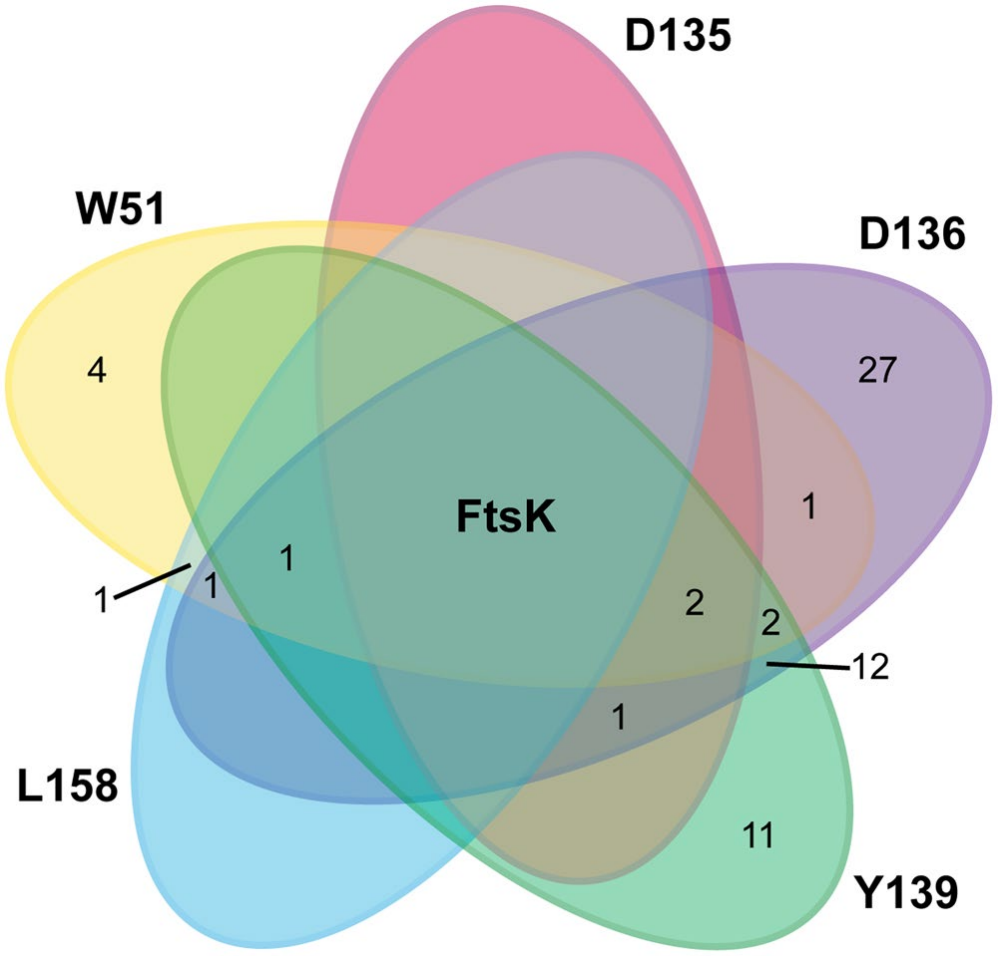
Distribution of protein groups detected by mass spectrometry of potential FtsK_N_ interaction partners. FtsK_N_* variants W51*, D135*, D136*, Y139* and L158* were purified from cultures treated with (+) and without (−) UV light, and digested with chymotrypsin. The resulting peptides were analysed by LC-MS/MS on a Q-Exactive Orbitrap mass spectrometer (ThermoFisher) to detect cross-linked proteins. Numbers indicate proteins exclusively detected in UV-treated samples (absent in untreated and WT controls) and identified with a minimum of 2 unique peptides.

Whole cells expressing each of the five FtsK_N_* variants were irradiated with long wavelength UV light to capture endogenous protein interaction partners *in vivo*. The irradiated cells were lysed, and the protein complexes were purified from the membrane fraction under stringent conditions by immobilized metal affinity chromatography (IMAC). FtsK_N_ and corresponding periplasmic interaction partners in each sample were then identified by LC-MS/MS. In total, 63 different proteins were identified, distributed across all five cross-linked samples (minimum 2 unique peptides; Fig. 2, Supplementary Table S1). All proteins were detected in UV-treated samples only and were absent from untreated and WT controls. Of the proteins identified, 92% were found in at least one cross-linked sample at or near the functional loop of FtsK_N_ (residues D135, D136 or Y139), suggesting this region may be critical for the maintenance of multiple protein contacts during growth and division.

**Figure 3:**
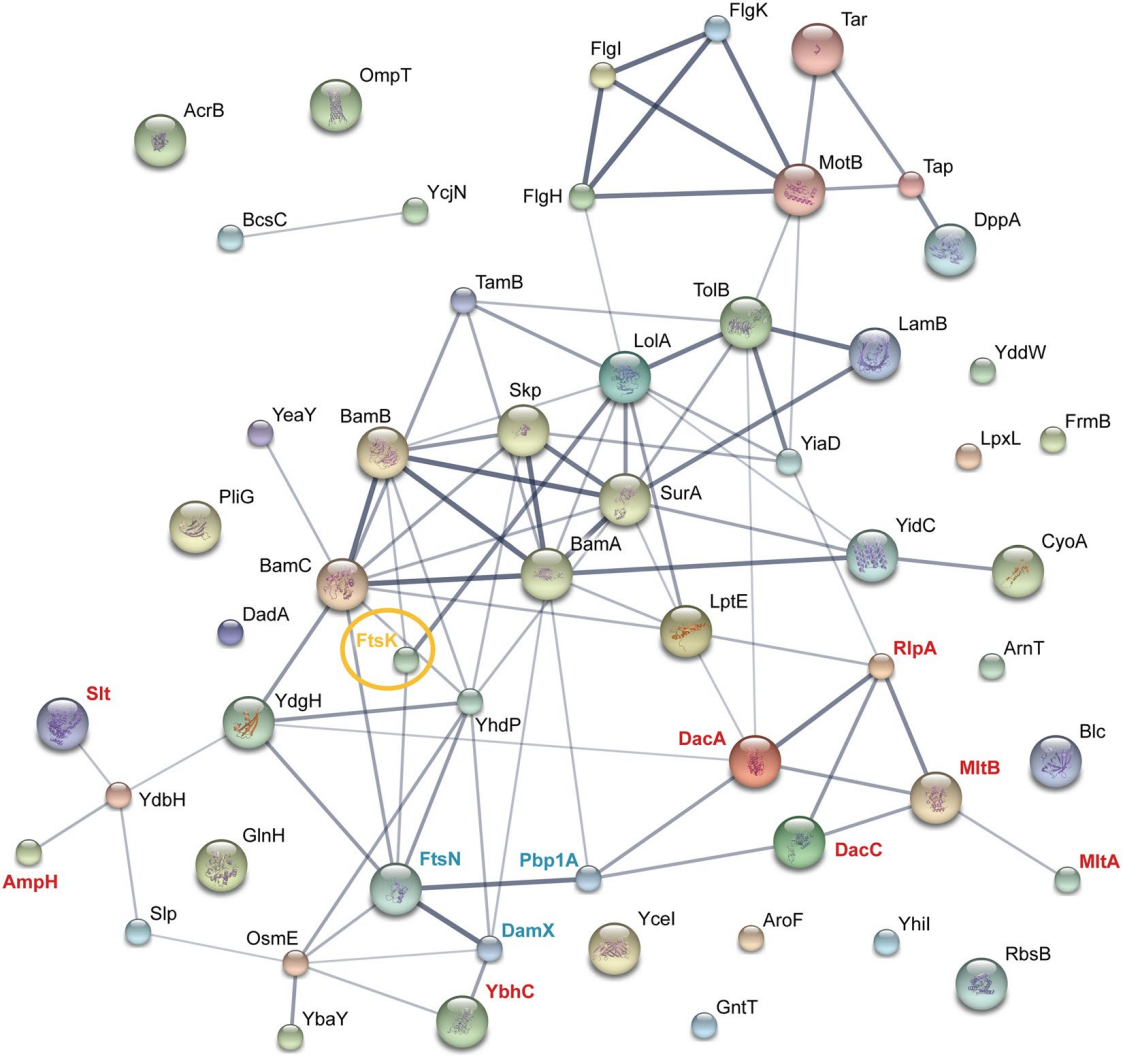
STRING protein interaction network of potential FtsK_N_ interaction partners identified by mass spectrometry. Proteins shown were identified exclusively in UV treated samples of FtsK_N_* variants W51*, D135*, D136*, Y139* and L158* (absent in untreated and WT controls). Interactions were determined using STRING 10.0^65^. Lines indicate known or predicted protein-protein interactions, with thicker lines indicating higher levels of confidence (minimum interaction score, 0.4). Models of known protein structures are shown inside the corresponding protein spheres. Blue text highlights proteins involved in elongation and cell division, red text indicates proteins involved in general peptidoglycan modification. FtsK is circled and highlighted in yellow.

Our network of potential FtsK_N_ interaction partners includes multiple protein groups of highly related function, including cell division proteins FtsN and DamX, and elongasome protein PBP1a (Fig. 3, Supplementary Table S1). Interestingly, a large proportion of the proteins detected are involved in cell wall organization and modification. As shown previously, disruption of the functional periplasmic loop of FtsK_N_ leads to uncoupling of septation events, and improper invagination of the peptidoglycan and outer membrane layers of the cell envelope^25^. This suggests that the function of FtsK in division might be to couple cell envelope septation events and transition the cell from elongation to septation. Therefore, to carry out its role as this essential checkpoint, it stands to reason that the periplasmic loops of FtsK_N_ would be in direct contact with the peptidoglycan synthesis machinery.

The use of mass spectrometry to identify purified protein complexes has notable limitations with respect to the specificity and sensitivity of the data provided. While affinity chromatography affords the ability to purify protein complexes that contain FtsK_N_, it cannot guarantee complete removal of other proteins or native complexes in tight association with the FtsK_N_ cross-linked adduct. In conjunction with the attomolar sensitivity common with mass spectrometry analysis^48^, identification of even the smallest amount of contaminating proteins is unavoidable. Taken together with such a diverse network of proteins isolated, it is unlikely that all 63 proteins identified truly interact with FtsK *in vivo*. As such, further biochemical and *in vivo* verification of the potential FtsK_N_ interaction partners is required.

To begin validation, we narrowed our list of candidate proteins by several criteria (Supplementary Table S1). First, proteins were ranked based on the average spectrum count and total number of unique peptides detected across all samples processed by LC-MS/MS to give a basic measure of protein abundance. While the LC-MS/MS method used was not quantitative with respect to absolute protein abundance, spectral counting, which counts and compares the number of fragment spectra identifying peptides of a given protein, can be used as a semi-quantitative, label free method for estimating protein abundance^49^. Ranking the protein list by the total number of unique peptides identified (i.e., the number of different amino acid sequences that are attributed to a single protein) also allowed us to roughly filter the putative FtsK_N_ interaction partners by the confidence of identification. By this parameter, a greater number of unique peptides would denote increased confidence that the protein has been correctly identified in the sample. Second, proteins were also classified based on the total number of cross-linked samples in which they were detected. Finally, to identify partners critical for the checkpoint function of FtsK_N_, we focused on proteins identified in all cross-linked samples at or near the functional periplasmic region of FtsK (residues D135, D136 and Y139). Following these criteria, septal peptidoglycan binding protein RlpA was chosen for further analysis because it had the highest average spectrum count and most unique peptides among proteins detected in 4 of the 5 cross-linked samples and in all cross-linked samples at or near the functional periplasmic region of FtsK.

### *In vitro* analysis of novel protein interaction between FtsK_N_ and RlpA

**Figure 4:**
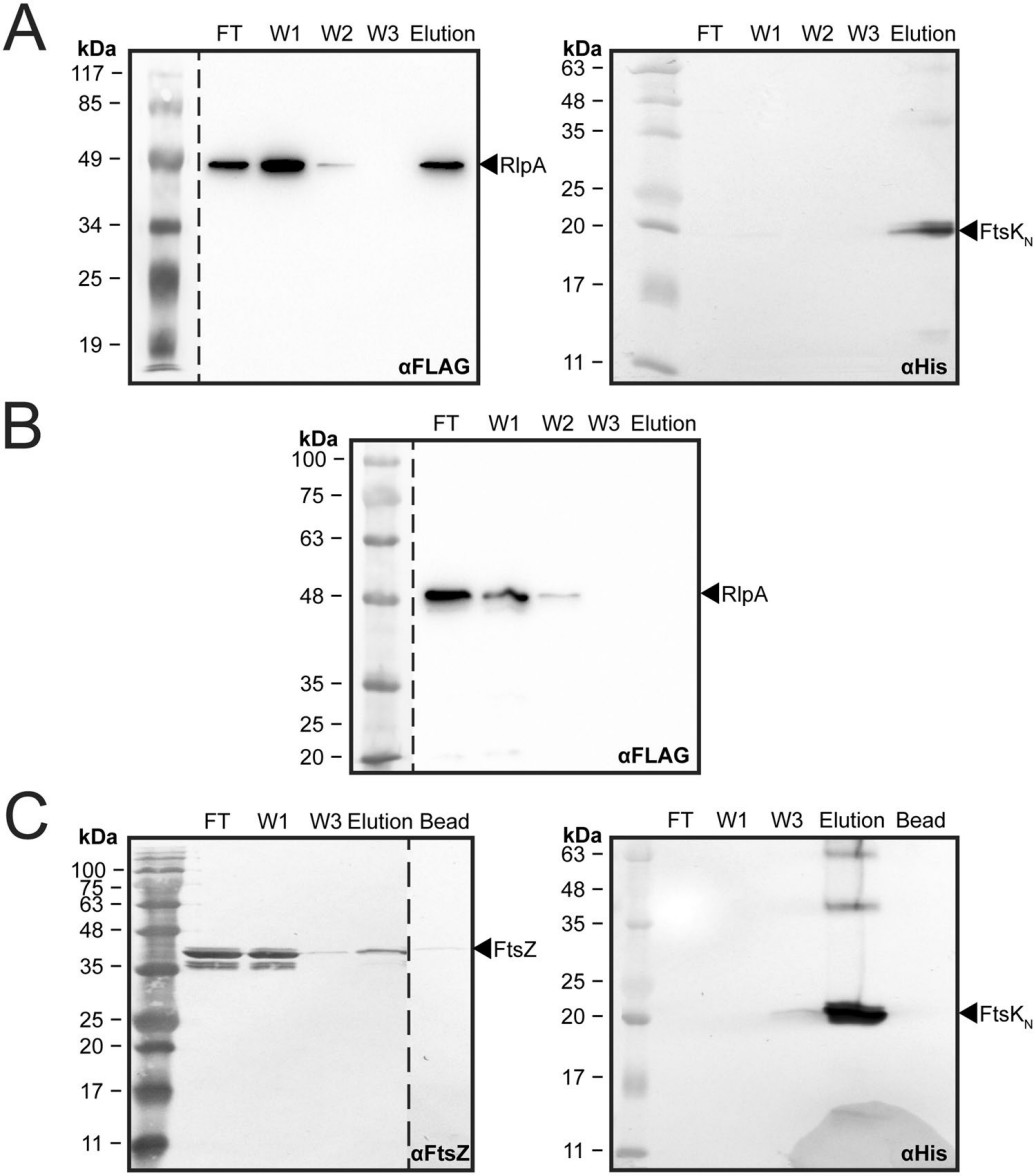
*In vitro* analysis of interaction between FtsK_N_ and RlpA. (**A**) Representative Western blots of pull-down assay between FLAG-RlpA (prey, *left panel*) and His_10_-FtsK_N_ (bait, *right panel*). Purified, immobilized His_10_-FtsK_N_ was incubated with 320 µg of purified FLAG-RlpA, washed to remove unbound protein and subsequently eluted to assess its interaction with RlpA by Western blot. (**B**) Western blot of FLAG-RlpA incubated with empty IMAC resin (negative control). (**C**) Representative Western blot of pull-down assay between FtsZ (prey, *left panel*) and His_10_-FtsK_N_ (bait, *right panel*). Purified, immobilized His_10_-FtsK_N_ was incubated with 250 µg of purified FtsZ, washed to remove unbound protein and subsequently eluted to assess its interaction with FtsZ. ‘*Bead*’ lane represents the elution fraction of FtsZ incubated with empty IMAC resin (negative control). For all blots, the primary antibodies are indicated in the bottom right corner. In all panels: *‘FT’* – flow through; *‘W1*, *W2*, *W3’* – wash fractions 1, 2, and 3; *‘Elution’* – elution with 1 M imidazole. Samples detected on separate blots are delineated by the dotted lines, and all were developed using the parameters outlined in the ‘Methods’ section. The representative molecular weight ladders in the anti-FLAG^®^ blots of panels A and B were captured by taking a reference image of the undeveloped blots.

Confirmation of direct interaction between FtsK_N_ and RlpA was achieved by an *in vitro* pull-down assay using His_10_-FtsK_N_ and a FLAG®-tagged soluble derivative of RlpA (FLAG-RlpA). Purified FLAG-RlpA was incubated with purified, IMAC resin-bound FtsK_N_ and successively washed to remove unbound protein. Upon elution of His_10_-FtsK_N_, RlpA and FtsK_N_ were detected in the elution fraction by Western blot analysis (Fig. 4A). To verify accuracy of the pull-down assay, RlpA was also incubated with IMAC resin in the absence of FtsK_N_ as a negative control and was not detected in the elution fraction (Fig. 4B). Thus, this assay indicated direct interaction between FtsK_N_ and RlpA. Additionally, purified FtsZ was used as a positive control of a known FtsK_N_ interaction partner. FtsZ was successfully detected in the elution fraction at an increased proportion when FtsK_N_ was bound to the IMAC resin (Fig. 4C, *Elution*), in comparison to the empty bead control (Fig. 4C, *Bead*), further verifying that the assay used can accurately detect the presence of a direct protein interaction with FtsK_N_ and supports identification of a novel protein interaction between FtsK_N_ and RlpA *in vitro*.

### Deletion of *rlpA* from LP11-1 (*ftsK44*) partially suppresses cellular filamentation

Although recent evidence has suggested that the septal peptidoglycan binding protein RlpA is a lytic transglycosylase involved in cell-cell separation in *P*. *aeruginosa*^37^, the exact function of RlpA in *E*. *coli* remains elusive. While several groups have attempted to uncover the role of RlpA in *E*. *coli* through the use of gene deletion mutants and enzymatic assays to probe the impact of purified RlpA on peptidoglycan degradation^34,35,37^, no study to date has been able to visualize any division-related phenotype for ∆*rlpA* mutants nor show enzymatic activity of the protein. In *E*. *coli*, *rlpA* is immediately upstream of the gene encoding penicillin binding protein 5 (*dacA*)^50^. Given deletion of *dacA* has been previously shown to suppress the loss of functional FtsK in temperature-sensitive *ftsK44* strains of *E*. *coli*^20^, we reasoned that a unique division-related phenotype might also be observed if *rlpA* was deleted in conjunction with the loss of FtsK.

Therefore, we deleted *rlpA* in the temperature-sensitive *E*. *coli* strain LP11-1 (*ftsK44*) to generate LP11-1 ∆*rlpA* (Supplementary Fig. S2) and assessed the impact of this deletion on cell growth and morphology. We also created an *rlpA* knockout in the *E*. *coli* strain W3110 to compare the impact of *rlpA* deletion in an otherwise WT background. In both cases, the gene deletion was created within the boundaries of the *rlpA* open reading frame to minimize disruption of the surrounding genes or potential promoter sequences and minimize polar effects.

**Figure 5:**
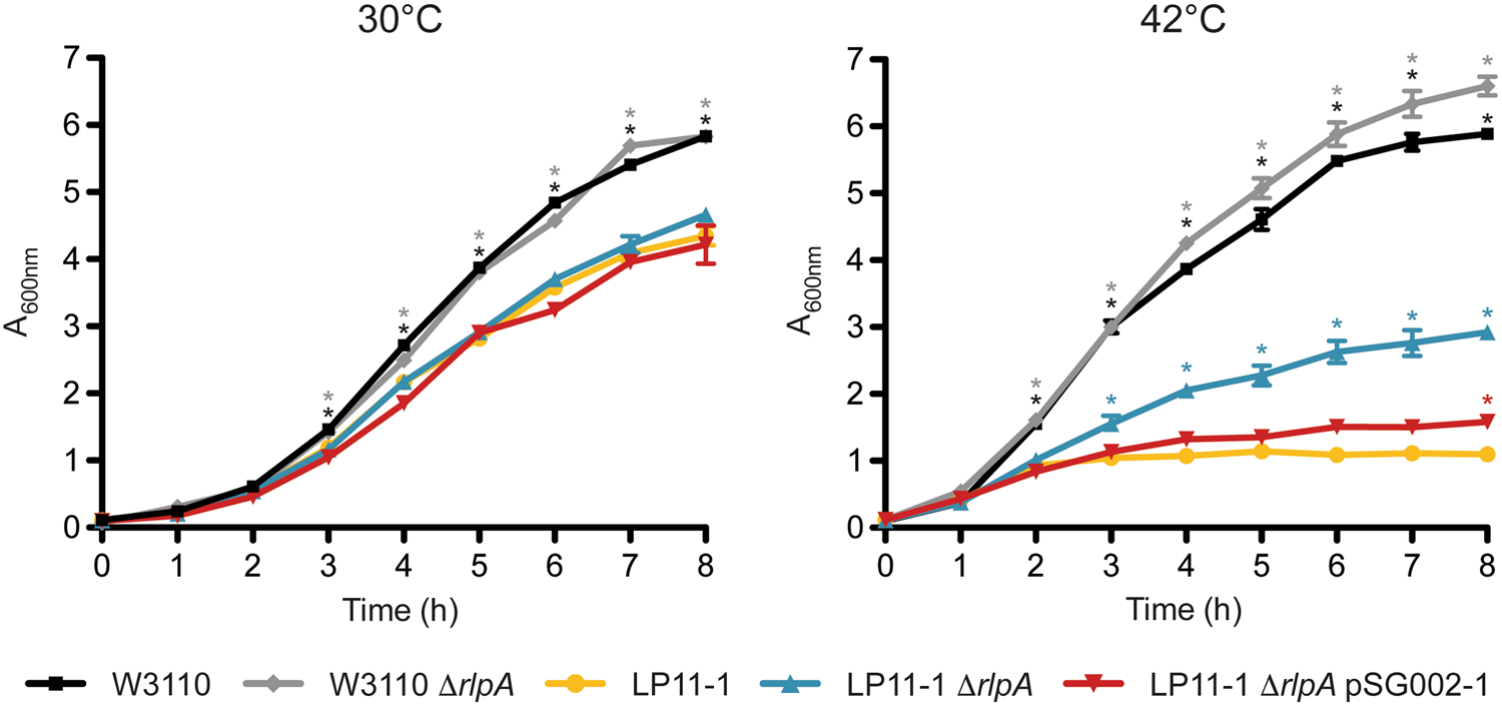
Deletion of *rlpA* partially alleviates growth inhibition of the temperature-sensitive *E*. *coli* strain LP11-1 (*ftsK44*). Growth curves of Δ*rlpA* knockout (W3110 Δ*rlpA* and LP11-1 Δ*rlpA*) and complemented (LP11-1 Δ*rlpA* pSG002-1) *E*. *coli* strains grown at permissive (30 °C) and nonpermissive (42 °C) temperatures (*n* = 3 per strain per temperature). The A_600_ of two technical replicates of each culture were sampled every hour using a SmartSpec^™^ Plus Spectrophotometer (Bio-Rad). In both panels, *E*. *coli* strains W3110 and LP11-1 were analyzed as WT controls. Pairwise comparison of growth differences was made using a one-way analysis of variance with Tukey-Kramer multiple comparison post-tests (*p < 0.05 *versus* LP11-1 at the same time point).

**Figure 6:**
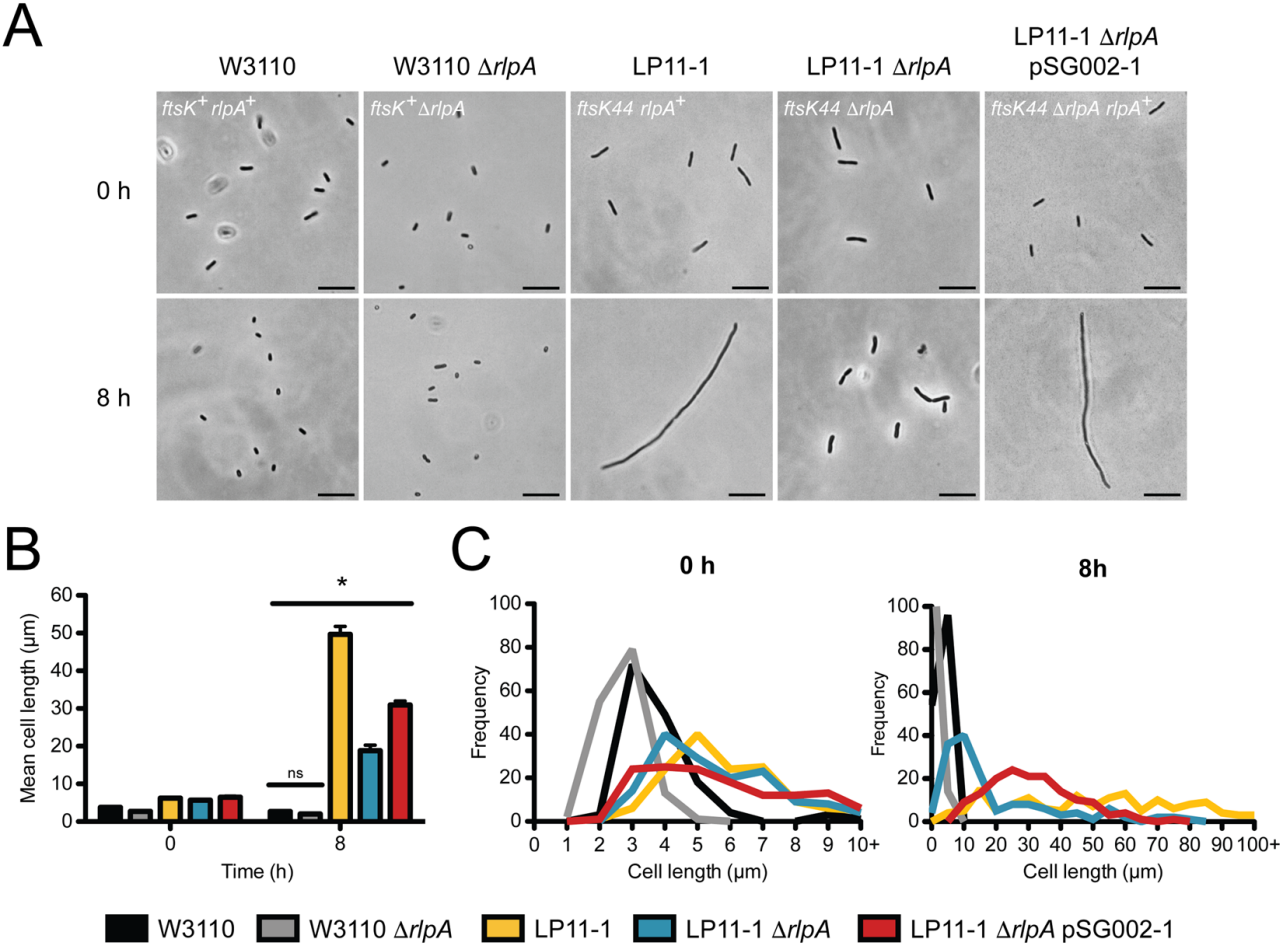
Deletion of *rlpA* restores normal cell morphology of the temperature-sensitive *E*. *coli* strain LP11-1 (*ftsK44*). (**A**) Representative phase contrast micrographs, (**B**) mean cell length, and (**C**) cell length distribution of Δ*rlpA* knockout (W3110 Δ*rlpA* and LP11-1 Δ*rlpA*) and complemented (LP11-1 Δ*rlpA* pSG002-1) *E*. *coli* strains grown at 42 °C. In all panels, *E*. *coli* strains W3110 and LP11-1 were analyzed as WT controls. Micrographs depict typical cells of each strain after 0 or 8 h of growth at 42 °C. The genotype of each strain is indicated in the upper left corner of each micrograph (*bar*, 10 µm). Cells were measured for cell length using the ImageJ program and are reported as mean cell length (µm) ± S.E. (*n* = 150 cells per strain per time point). Pairwise comparisons of cell lengths were made using a one-way analysis of variance with Tukey-Kramer multiple comparison post-tests (*p < 0.001).

To probe the impact of *rlpA* deletion in both the presence and absence of functional FtsK, we completed growth and morphology analysis of the LP11-1 ∆*rlpA* and W3110 ∆*rlpA* strains at both permissive (30 °C) and nonpermissive (42 °C) temperatures, respectively (Figs 5 and 6). Although the WT *E*. *coli* strain W3110 grew more rapidly and to a higher absorbance than the temperature-sensitive strain LP11-1 (*p* < 0.05) at both temperatures, deletion of *rlpA* only had a significant impact on growth in the absence of functional FtsK (i.e., in the LP11-1 background during growth at 42 °C) (Fig. 5). In this case, *rlpA* deletion significantly alleviated the severe growth defect caused by the loss of FtsK, and partially suppressed the cellular filamentation typically associated with the temperature-sensitive *ftsK44* mutation (Fig. 6). This partial restoration of growth and WT cell morphology could be negated by complementation of the LP11-1 ∆*rlpA* strain with a plasmid-encoded copy of *rlpA* (encoded on plasmid pSG002-1), suggesting that the combined loss of functional FtsK and RlpA was responsible for the observed phenotype.

## Discussion

Despite clear evidence that FtsK is essential during division, the limited amount of biochemical data currently available regarding the protein contacts it makes, or its biological function, gives only a crude picture of its role during division. With the cross-linking and deletion mutant data presented herein, we have uncovered an FtsK_N_ interactome that again points to a role for FtsK in cell envelope remodeling during division.

The use of site-specific cross-linking by the photoactivatable amino acid *p*Bpa in whole cells allows us to study the putative protein complexes FtsK_N_ forms in its natural environment, with minimal alterations to the full-length proteins involved. While this technique allowed us to identify a large number of potential FtsK_N_ interaction partners (Fig. 2, Supplementary Table S1), limitations regarding the specificity and high sensitivity of our LC-MS/MS approach makes further biochemical investigation of each protein as a direct FtsK_N_ interactor necessary. Regardless, the list of proteins identified in our cross-linking analysis provides further insight into the role of FtsK in cell division, as the majority of proteins detected could be grouped into several small functional groups, with a high degree of known interaction among them (Fig. 3). In particular, this study is the first to report a confirmed, direct interaction between the OM lipoprotein RlpA and an essential member of the divisome (Fig. 4). The implication of this interaction is difficult to interpret in the absence of definitive evidence on the function of RlpA in *E*. *coli*. However, general localization data in *E*. *coli*^34–36^ and recent biochemical evidence on the enzymatic activity of RlpA in *P*. *aeruginosa*^37^ highlights the potential significance of this novel interaction.

Of all SPOR-domain containing proteins, RlpA is the most highly conserved across bacterial species^37^. This would suggest that RlpA plays an important role in bacteria. Based on its OM localization and ability to preferentially bind septal peptidoglycan^34–36^, one role for RlpA could be to physically link the OM to the peptidoglycan and IM during constriction. In this capacity, the interaction between RlpA and FtsK would function similarly to the Tol-Pal interaction. In *E*. *coli*, the trans-envelope Tol-Pal complex localizes to the site of division and helps to coordinate invagination of the OM with both the peptidoglycan and IM cell envelope layers^51^. Intriguingly, TolB was identified as a potential FtsK interaction partner by our mass spectrometry analysis (Fig. 3, Supplementary Table S1). Cells lacking an intact Tol-Pal system show delayed OM invagination during cell constriction^51^, which poses an attractive link to the inhibition of OM and peptidoglycan invagination we first observed upon the expression of non-functional FtsK_N_ variants^25^. Interaction between the periplasmic loops of FtsK_N_ and the Tol-Pal complex would create a link between the IM and OM that could facilitate coordinated invagination of the entire cell envelope during division. This connection might then account for the cell void phenotype seen upon the expression of non-functional periplasmic loop mutants of FtsK_N_^25^, as disruption of this interaction would directly uncouple IM division from OM invagination. This functional redundancy might also explain why null mutants of *rlpA* in WT *E*. *coli* strains have no morphological defects, as the loss of the OM-IM contact made between RlpA and FtsK upon deletion of RlpA could be accommodated by an intact interaction maintained by FtsK with the Tol-Pal complex. However, this scenario does not fully account for the restoration of growth seen in the absence of both RlpA and functional FtsK_N_ (Fig. 5), indicating RlpA must play a larger role in division beyond simply bridging all three cell envelope layers.

A second role for RlpA may be in balancing lateral growth of the cell *versus* septal peptidoglycan synthesis. In addition to its septal localization, RlpA is also found at distinct foci along the cell cylinder^34^. Together with the fact that *rlpA* is encoded immediately downstream of two proteins involved in cell elongation, *pbpA* (encoding PBP2) and *rodA*^50^, it is proposed that RlpA might function as part of the elongation machinery, in addition to the divisome. Interaction between FtsK and RlpA would then represent another link between these two macromolecular complexes, and potentially facilitate the transition between elongation and septation in *E*. *coli*. The restoration of growth seen upon the loss of both RlpA and functional FtsK suggests that their interaction may be critical in suppressing septation until sufficient elongation and proper assembly of the divisome occurs.

Perhaps the most intriguing aspect of the interaction between RlpA and FtsK_N_ is its parallel with the functional interaction between FtsK and the carboxypeptidase DacA (PBP5). DacA catalyzes the removal of the terminal d-alanine from peptidoglycan side chains during cell wall synthesis^2,52^. Interestingly, DacA was also detected as a potential FtsK_N_ interaction partner in our cross-linking analysis (Fig. 3, Supplementary Table S1). Deletion of *dacA* from *E*. *coli* carrying the temperature-sensitive *ftsK44* allele has been shown to largely suppress filamentation of bacteria grown at a nonpermissive temperature (42 °C)^20^. This is in direct correlation with the phenotype we observe upon the deletion of *rlpA* from the same strain of *E*. *coli* (Fig. 6). In addition, and perhaps not surprisingly, *rlpA* also resides on the chromosome immediately upstream of *dacA*^50^. Given their proximity and evidence that RlpA is also involved in peptidoglycan remodeling^37^, it would not be unreasonable to estimate that the deletion of either of these genes would have a similar impact on the cell in the absence of functional FtsK. In the case of *dacA*, deletion of this gene is only able to compensate for the temperature-sensitive growth defect and not complete loss of FtsK^53^, suggesting that under these circumstances cells might be able to restore the function of the mutated FtsK44 protein rather than completely bypass its function. How this occurs is unclear, but we postulate that if DacA and FtsK form a direct protein interaction, deletion of DacA would free FtsK to form protein contacts with additional proteins that could stabilize its structure and restore its function. In our study, it would be pertinent to note that this may also be the case with the deletion of *rlpA*, and that further investigation into the ability of an *rlpA* deletion to permit the complete deletion of *ftsK* is necessary.

In addition to the verified interaction between FtsK_N_ and RlpA described above, we were also able to detect several proteins involved in growth and division (e.g., divisome proteins FtsN and DamX, and elongasome protein PBP1a). These proteins represent novel potential FtsK interactors, as none of the proteins are previously reported in the literature as FtsK interaction partners. Both FtsN and DamX are SPOR-domain containing proteins that bind peptidoglycan and are targeted to the septum during division^34–36^. Specifically, FtsN is an essential component of the divisome that is responsible for activation of cell constriction during late stages of division^54–56^. In contrast, while DamX is an early recruit to the septum and interacts with several divisome proteins, it is not essential for cell division and currently has no known function^34,35,57^. During elongation, PBP1a acts as a bifunctional transglycosylase/transpeptidase that modifies and synthesizes the growing peptidoglycan layer^58^. Given limited knowledge about the functions of both FtsK_N_ and DamX during division, the significance of an interaction between these two proteins is difficult to interpret. However, the interactions of FtsK with FtsN and PBP1a provide an intriguing link between the early and late stages of division, and the switch from cell elongation to septation in *E*. *coli*. The formation of a tripartite complex with FtsK_N_, FtsN, and PBP1a would physically link the elongasome with the divisome in the periplasm and allow for the direct transmission of a cell signal between these two essential complexes. Completion of cell elongation by the elongasome would be sensed through the interaction between PBP1a and FtsK_N_. In turn, this signal could be integrated with a signal for the completion of divisome formation and proper chromosome segregation sensed by FtsK, and then passed to FtsN through direct interaction of these two proteins to trigger constriction. While this report is the first to suggest direct interaction between FtsK and FtsN in *E*. *coli*, these proteins have been shown to interact in the Gram-negative bacterium *Neisseria gonorrhoeae*^59^. Together, these interactions shed light onto a potential mechanism of action for FtsK_N_ in cell envelope remodeling during both growth and division.

Based on prior biochemical evidence, it is important to note that essential cell division proteins previously shown to interact with FtsK by bacterial two-hybrid analysis, including IM proteins FtsQ, FtsL, and FtsI^27,31,32^, were not detected in our cross-linking analysis. Given we have elected to only probe the periplasmic face of FtsK_N_, it is possible that these proteins interact with FtsK at locations distinct from our sites of inquiry, namely the transmembrane or cytoplasmic domains of the protein. Alternatively, it is possible that FtsK does not interact directly with these proteins, but rather they are bridged by a third interacting partner that would have confounded previous bacterial two-hybrid results. For example, FtsI has been shown to form a complex with the OM lytic transglycosylases MltA and MltB^60,61^; therefore, our detection of these two proteins by LC-MS/MS (Fig. 3, Supplementary Table S1) might explain formation of a tertiary complex between FtsK and FtsI bridged by these lytic transglycosylases.

As a whole, the novel periplasmic FtsK_N_ interactome and verified interaction with RlpA described in this study provides further evidence for FtsK as a potential regulator of cell envelope remodeling during division. While the identity of the interactor responsible for the shift from cell elongation to septation remains unclear, we detected several proteins that could fulfill this role. In fact, it is most likely that a number of these proteins work in concert to accurately move the bacterium through its growth cycle. As with all of the potential FtsK_N_ interaction partners identified, further biochemical verification of the specificity and validity of these interactions is needed to characterize each protein as a true interactor. Further investigation into the impact each interaction with FtsK has on both growth and division will undoubtedly enhance our understanding of both of these essential processes.

## Methods

### Bacterial strains, plasmids, and growth conditions

Bacterial strains and plasmids used in this study are listed in Table 1. *E*. *coli* W3110, DH5α and Lemo21 cultures were grown at 37 °C in lysogeny broth (LB) (BD Biosciences) in a rotary shaker at 200 rpm. Media were supplemented with 150 µg/mL ampicillin, 30 µg/mL chloramphenicol or 50 µg/mL kanamycin where appropriate for plasmid-carrying strains. *E*. *coli* LP11-1 cultures were grown in Complementation Media (1% [w/v] tryptone, 0.5% [w/v] yeast extract, and 1% [w/v] NaCl [Fisher]) supplemented with 15 µg/mL tetracycline, as well as appropriate antibiotics for plasmid selection where applicable. Cultures were grown at 30 °C or 42 °C, as described previously^25^. Maintenance of the kanamycin resistance cassette in the Δ*rlpA* knockout strains was achieved by the addition of 25 µg/mL kanamycin to all overnight cultures. To assess the impact of deletion of *rlpA* on cell growth and morphology, overnight cultures of LP11-1 Δ*rlpA* and W3110 Δ*rlpA* were each diluted to an A_600_ of 0.1 (SmartSpec™ Plus Spectrophotometer; Bio-Rad) in two separate 25-mL aliquots of fresh Complementation Media and grown at 30 °C or 42 °C for 8 h (*n* = 3 per strain per temperature). The A_600_ of two technical replicates of each culture were sampled every hour and cells were imaged by phase contrast microscopy (Leica DM2000 LED, ProgRes CT3 camera; Jenoptik AG). Cell length was assessed for 150 random cells from each culture using the ImageJ program (version 1.46r, National Institutes of Health) and is reported as mean length ± S.E. Statistical analysis for cell growth (A_600_) and mean length were completed using one-way analysis of variance with Tukey-Kramer multiple comparison post-tests by Prism 5 software (GraphPad Software, Inc.) using a level of significance of α = 0.05 for all tests. *E*. *coli* K12 W3110 and LP11-1 were also grown at 30 °C and 42 °C as described above for wild-type (WT) controls. Deletion of *rlpA* was complemented in *trans* by expression of *FLAG-rlpA* from plasmid pSG002-1 in strain LP11-1 Δ*rlpA* using 80 ng/mL anhydrotetracycline (ATc).

**Table 1:** Bacterial strains and plasmids.

Strain or plasmid	Description	Source or Reference
***E***. ***coli*** **strain**		
W3110	*rph-1*IN (*rrnD*-*rrnE*)	Coli Genetic Stock Center
Lemo21 (DE3)	*fhuA2* [*lon*] *ompT gal* (*λDE3*) [*dcm*] Δ*hsdS*/ pLemo(Cam^R^) λ DE3 = λ *sBamHIo* Δ*EcoRI-B int*::(*lacI*::*PlacUV5*::*T7 gene1*) *i21* Δ*nin5* pLemo = pACYC184-*PrhaBAD-lysY*	New England Biolabs
DH5α	F−Φ80*lacZ*ΔM15Δ (*lacZYA-argF*) U169 *rec*A1 *end*A1 *hsd*R17	Invitrogen
	(rK−, mK+) *pho*A *sup*E44 λ-*thi*-1 *gyr*A96 *rel*A1	
LP11-1	W3110 *ftsK44 aroA*::Tn10	K. Young^69^
LP11-1 Δ*rlpA*	W3110 *ftsK44 aroA*::Tn10 Δ*rlpA*::*kan*	This study
W3110 Δ*rlpA*	W3110 Δ*rlpA*::*kan*	This study
**Plasmids**		
pEVOL-pBpF	Expression vector encoding tRNA/aminoacyl-tRNA synthetase pair derived from *Methanococcus jannaschii* for *in vivo* incorporation of the photo-crosslinker *p*-benzoyl-l-phenylalanine (*p*Bpa) into proteins in *E*. *coli*; Cam^R^	Addgene (no. 31190)^43^
pKD4	Template plasmid for λ red mediated gene deletion; Kan^R^	^68^
pKD46	Red recombinase expression plasmid under the control of P_ara_; Amp^R^	^68^
pBAD24	Protein expression vector under the control of P_ara_; Amp^R^	Addgene
pWQ743	Protein expression vector under the control of P_tet_; Amp^R^	C. Whitfield^41^
pET28a	Protein expression vector under the control of P_lac_; Kan^R^	EMD Millipore
pWQ572	Protein expression vector under the control of P_tet_; Cam^R^	C. Whitfield^41^
pAB006-2	pBAD24 derivative encoding amino acids 1-220 of FtsK (His_10_-FtsK_N(220)_) from *E*. *coli*	^25^
pAB008-1	pWQ743 derivative encoding amino acids 1-220 of FtsK (His_10_-FtsK_N(220)_) from *E*. *coli*	This study
pSG001-1	pET28a derivative encoding amino acids 18-362 of RlpA with an N-terminal FLAG-tag (FLAG-RlpA) from *E*. *coli*	This study
pSG002-1	pWQ572 derivative encoding amino acids 18-362 of RlpA with an N-terminal FLAG-tag (FLAG-RlpA) from *E*. *coli*	This study

The abbreviations used are as follows: Amp, ampicillin; Cam, chloramphenicol; Kan, kanamycin.

### Plasmid construction

An oligonucleotide encoding the N-terminal 220 amino acids of FtsK (FtsK_N(220)_) with an N-terminal decahistidine tag was cloned into the ATc-inducible expression vector pWQ743 to produce pAB008-1, as described previously^25^. For over-expression and purification of RlpA, an oligonucleotide encoding a soluble derivative of RlpA containing an N-terminal FLAG®-tag in place of its type II signal sequence (FLAG-RlpA) was cloned into expression vector pET28a as described previously^25^, using custom primers AMB006Fa (5′-TGGACCATGGATTATAAAGATGATGATGATAAATCCAGTACAAGCGATGATGGTCAGC-3′) and AMB006R (5′-AAGGTCAAGCTTACTTTACTGCGCGGTAGTAATAAATGACTGTAATTGGGCTTC-3′) to produce pSG001-1. Similarly, for complementation of the Δ*rlpA* knockout strains, the oligonucleotide encoding FLAG-RlpA was also cloned into the ATc-inducible expression vector pWQ572 using the above primers, producing pSG002-1. All constructs were verified by DNA sequencing (Genomics Facility, Advanced Analysis Center, University of Guelph).

### Site-directed mutagenesis

To capture FtsK_N_ protein interaction partners, five site-specific amber stop codon variants of FtsK_N_ (FtsK_N_*) were generated by site-directed mutagenesis. A single amber codon (TAG) was introduced into the sequence of *ftsK*_*N*_ corresponding to the periplasmic amino acid positions Trp-51, Asp-135, Asp-136, Tyr-139 or Leu-158 using a QuikChange Lightning site-directed mutagenesis kit (Stratagene). All primers used for mutagenesis and resulting plasmids are shown in Table 2. All single amber codon variants were confirmed by DNA sequencing (Genomics Facility, Advanced Analysis Center, University of Guelph).

**Table 2:** Oligonucleotide pairs used for site-directed mutagenesis.

Mutation	Sequence of mutagenic oligonucleotide (5′ to 3′)^a^	Plasmid
W51*	CGGACCCCAGC**TAG**TCGCAAACGGC	pAB008-W51*
	GCCGTTTGCGA**CTA**GCTGGGGTCCG	
D135*	CTGGCGGCAATCAACGCT**TAG**GATATCTGGTATTTTCGG	pAB008-D135*
	GGCAAAATACCAGATATC**CTA**AGCGTTGATTGCCGCCAG	
D136*	GCGGCAATCAACGCTGAC**TAG**ATCTGGTATTTTGCCTCC	pAB008-D136*
	GGAGGCAAAATACCAGAT**CTA**GTCAGCGTTGATTGCCGC	
Y139*	CTGACGATATCTGG**TAG**TTTGCCTCCGGTGGCG	pAB008-Y139*
	CGCCACCGGAGGCAAA**CTA**CCAGATATCGTCAG	
L158*	CACTACGCTACAACCACTG**TAG**CACAGTAGCGGGGGAACTA	pAB008-L158*
	TAGTTCCCCCGCTACTGTG**CTA**CAGTGGTTGTAGCGTAGTG	

^a^Base changes are underlined and in boldface.

### *In vivo* UV cross-linking

For *in vivo* cross-linking of FtsK_N_ interacting proteins, the photoactivatable amino acid *p*-benzoyl-l-phenylalanine (*p*Bpa) was incorporated into FtsK_N_ by a mutant tRNA/tRNA synthetase pair from *Methanococcus jannaschii* that has been engineered to recognize the amber stop codon (TAG)^43^. Cross-linking of whole bacterial cells expressing site-specific amber stop codon variants of FtsK_N_ (FtsK_N_*) in the presence of *p*Bpa and the mutant tRNA/tRNA synthetase (encoded on plasmid pEVOL-pBpF) was performed as described by Nickerson *et al*.^41^, with minor modifications. Briefly, overnight cultures of *E*. *coli* LP11-1 strains carrying FtsK_N_* variants were diluted to an A_600_ of 0.1 (SmartSpec™ Plus Spectrophotometer; Bio-Rad) in 400 mL Complementation Media supplemented with ampicillin and chloramphenicol (75 μg/mL and 15 μg/mL, respectively), 1 mM *p*Bpa (Bachem), as well as 0.02% (w/v) l-arabinose to induce synthesis of the tRNA/tRNA synthetase pair from pEVOL-pBpF. FtsK_N_* variants were induced with 40 ng/mL ATc. Cultures were incubated under reduced lighting at 42 °C for 2 h in a rotary shaker at 200 rpm. Identical cultures grown in the absence of 1 mM *p*Bpa were prepared to assess the efficiency of *p*Bpa incorporation into full-length FtsK_N_. One-millilitre aliquots of cultures grown in the presence and absence of *p*Bpa were concentrated 1:100, mixed with 20 µL of 5 × Loading Buffer (250 mM Tris-HCl, pH 6.8, 10% [w/v] SDS, 30% [v/v] glycerol, 5% [v/v] β-mercaptoethanol, 0.1% [w/v] bromophenol blue), and boiled for 10 min in a covered beaker. Samples were analyzed by SDS-PAGE and Western blotting as described below.

To induce *in vivo* cross-linking of FtsK_N_* variants, cultures grown in the presence of *p*Bpa were harvested by centrifugation and washed twice in 10 mL sterile PBS. The resulting cell pellets were suspended into 10 mL phosphate-buffered saline (PBS) for every A_600_ 0.1 of the original culture (e.g., a culture with an A_600_ of 0.5 was suspended in 50 mL PBS) before splitting into two equal aliquots. Aliquot #1 was placed into a 6-well tissue culture plate on ice (5 mL suspension per well) and the whole cells were irradiated at 365 nm with a handheld UV lamp (Spectroline Model EN-180L; 2.5 cm from surface of the cell suspension) for 15 min. Aliquot #2 was covered in tinfoil and incubated on ice for 15 min as a non-crosslinked control. Both aliquots were harvested by centrifugation and the pellets were stored at −20 °C. All samples were protected from additional light exposure, where possible, during storage and purification of FtsK_N_. All samples were prepared in duplicate from biological replicate cultures. An additional culture expressing WT FtsK_N(220)_ was harvested and processed in parallel with cross-linked and non-crosslinked control cells as described below to account for contaminant proteins inherent to the purification of FtsK_N_.

Cross-linked (aliquot #1) and non-crosslinked control cells (aliquot #2) were thawed in 3 mL SDS Lysis Buffer (20 mM Tris-HCl, pH 7.0, 100 mM NaCl, 1% [w/v] SDS) and sonicated on ice for 1 min (10 s on, 10 s off; Misonix Ultrasonic Processor XL2020, Misonix, Inc.) to complete cell lysis. Samples were then diluted with 27 mL Purification Buffer (20 mM Tris-HCl, pH 7.4, 300 mM NaCl, 25 mM imidazole) to reach a final SDS concentration of 0.1% (w/v). Profinity Ni^2+^-charged immobilized metal affinity chromatography (IMAC) resin (Bio-Rad) was added to each sample at a bed volume of 100 µL and then incubated at 4 °C for 1.5 h with rocking. Cell lysate/resin mixtures were collected by centrifugation at 4,500 × *g* for 5 min at 4 °C and washed five times with 1 mL of Purification Buffer containing 0.1% (w/v) SDS before performing on-bead digestion for protein analysis.

### On-bead digestion and liquid chromatography tandem-mass spectrometry (LC-MS/MS)

WT FtsK_N_ and FtsK_N_* from cross-linked and non-crosslinked controls were subjected to on-bead proteolytic digestion with chymotrypsin as described by Park *et al*.^62^. Briefly, resin mixtures containing FtsK_N_ complexes were washed five times in 1 mL freshly prepared 50 mM ammonium bicarbonate (ABC) buffer (pH 8.0). Beads were then suspended in Denaturation Buffer (6 M urea/2 M thiourea in 10 mM HEPES, pH 8.0) and proteins were subsequently reduced by incubation at 50 °C for 30 min in Reduction Buffer (10 mM dithiothreitol in ABC buffer). Samples were then incubated in Alkylation Buffer (55 mM iodoacetamide in ABC buffer) for 30 min at room temperature in the dark. Reduced and alkylated proteins were diluted with ABC buffer and digested with 1 µg chymotrypsin (Princeton Separations) in the presence of 0.01% (w/v) ProteaseMAX surfactant (Promega) at 30 °C overnight. The IMAC resin was removed by centrifugation and digested peptides in the supernatant were lyophilized using a speed-vacuum concentrator. Samples were reconstituted in 1% trifluoroacetic acid in water and passed through a C18 stage tip to remove any trace detergent and salt from the purification process, and lyophilized.

To identify cross-linked proteins, samples were analyzed by liquid chromatography tandem-mass spectrometry (LC-MS/MS) on an Orbitrap analyzer (Q-Exactive, ThermoFisher) outfitted with a nanospray source and EASY-nLC nano-LC system (ThermoFisher). Lyophilized peptide mixtures were dissolved in 0.1% formic acid and loaded onto a 75 µm × 50 cm PepMax RSLC EASY-Spray column filled with 2 µm C_18_ beads (ThermoFisher) at a pressure of 800 Bar heated to 60 °C. Peptides were eluted over 90 min at a rate of 250 nL/min using a 0 to 42% Buffer A to Buffer B gradient (Buffer A: 0.1% formic acid; Buffer B: 80% acetonitrile, 0.1% formic acid).

Peptides were introduced by nano-electrospray into the Q-Exactive mass spectrometer (ThermoFisher). The instrument method consisted of one MS full scan (400–1500 *m/z*) in the Orbitrap mass analyzer with an automatic gain control (AGC) target of 1 × 10^6^, maximum ion injection time of 120 ms and a resolution of 70,000, followed by 10 data-dependent MS/MS scans with a resolution of 17,500, an AGC target of 1 × 10^6^, maximum ion time of 120 ms, and one microscan. The intensity threshold to trigger an MS/MS scan was set to 6.7 × 10^4^. Fragmentation occurred in the higher-energy collisional dissociation (HCD) trap with normalized collision energy set to 27. The dynamic exclusion was applied using a setting of 10 seconds.

Raw data files were loaded into PEAKS 7 software (Bioinformatics Solutions, Inc.) for peptide and protein analysis using the UniProtKB *E*. *coli* K12 database. The generated protein and peptide identifications were then visualized using Scaffold (version 4.4.8, Proteome Software Inc., Portland, OR). Proteins identified with >95% probability using the Peptide^63^ and Protein^64^ Prophet algorithms, and a minimum of 2 unique peptides in both biological replicates, were accepted as positive identifications. Proteins detected in the WT preparation of FtsK_N_ and non-crosslinked negative controls were used as an exclusion list for proteins found in the cross-linked samples. Proteins conclusively reported to have a cytoplasmic cellular localization were also excluded. Protein-protein interaction networks of all cross-linked proteins were built using STRING version 10.0^65^ using all available prediction methods and a minimum confidence level of 0.4.

### Expression and purification of His_10_-FtsK_N(220)_

Overnight cultures of *E*. *coli* Lemo21 cells carrying WT His_10_-FtsK_N(220)_ encoded by a pBAD24 derivative (plasmid pAB006-2) were diluted 1:50 into 250 mL LB media and grown at 37 °C for 1.5 h. l-arabinose was added to a final concentration of 0.2% (w/v) and incubated for another hour. Following induction, the entire culture was harvested by centrifugation (8,000 × *g*, 10 min, 4 °C) and cells were resuspended in 5 mL of Lysis Buffer (20 mM Tris-HCl pH 7.0, 100 mM NaCl, 4 mM EDTA, 40 µg/mL DNaseI, 40 µg/mL RNase A, and 300 µg/mL lysozyme). Samples were incubated at room temperature for 10 min with rocking, then sonicated for 1 min (10 s on, 10 s off) to complete cell lysis. The total membrane fraction was collected by ultracentrifugation at 120,000 × *g* for 1 h at 4 °C (Beckman L8-55 M ultracentrifuge, Ti70 rotor). Membranes were suspended in 3 mL Purification Buffer (20 mM Tris-HCl pH 7.4, 300 mM NaCl, 25 mM imidazole, 10% [v/v] glycerol) containing 2% lauryldimethylamine-oxide (LDAO) and 100 µL of Profinity Ni^2+^-charged IMAC resin (Bio-Rad) and were incubated at 4 °C for 1.5 h with rocking. Solubilized membrane/resin mixtures were collected by centrifugation at 14,000 × *g* for 1 min at 4 °C in microcentrifuge tubes and washed ten times with 1 mL of Purification Buffer containing 0.05% LDAO. Purified, resin-bound FtsK_N_ was then used during *in vitro* characterization of protein interaction between FtsK and RlpA by pull-down assay.

### Expression and purification of FLAG-RlpA

Overnight cultures of *E*. *coli* Lemo21 cells carrying soluble FLAG-RlpA encoded by a pET28a derivative (plasmid pSG001-1) were diluted 1:100 into 1 L LB media and grown at 37 °C for 1 h before transferring to 30 °C for an additional hour of growth. Isopropyl-β-d-thiogalactoside (IPTG) was added to 1 mM and the culture was incubated for an additional 3 h at 30 °C. Following induction, the entire culture was harvested by centrifugation (8,000 × *g*, 10 min, 4 °C) and resuspended in 25 mL of Lysis Buffer before lysing by three passes through a French pressure cell (operating at 1000 psi). To remove cellular debris, samples were centrifuged at 8,000 × *g* for 10 min at 4 °C. The resulting supernatant was collected and ultracentrifuged at 120,000 × *g* for 1 h at 4 °C (Beckman L8-55 M ultracentrifuge, Ti70 rotor) to remove the membrane fraction. The resulting supernatant containing soluble FLAG-RlpA was added to 300 µL of anti-FLAG^®^ M2 affinity gel (Sigma-Aldrich) and incubated overnight at 4 °C with rocking. FLAG-RlpA/resin mixtures were collected by centrifugation at 100 × *g* for 2 min at 4 °C and washed five times with 1 mL of Wash Buffer (20 mM Tris-HCl pH 7.0, 100 mM NaCl). FLAG-RlpA was successively eluted in four washes of 250 µL Wash Buffer containing 100 µg/mL FLAG® peptide (Sigma-Aldrich). Eluted FLAG-RlpA was pooled and the total protein concentration was determined by a bicinchoninic acid protein assay as per manufacturer’s instructions (Thermo Scientific) using bovine serum albumin (BSA) as a standard.

### Pull-down assays

To assess the interaction between FtsK and RlpA, 320 µg of purified FLAG-RlpA was incubated with 100 µL of IMAC resin-bound His_10_-FtsK_N_ at 4 °C overnight with rocking. The beads were collected by centrifugation and washed three times in 1 mL of Wash Buffer containing 25 mM imidazole and 0.05% LDAO. Bound proteins were eluted in 240 µL of Wash Buffer containing 1 M imidazole and 0.05% LDAO, mixed with 60 µL of 5 × Loading Buffer (250 mM Tris-HCl, pH 6.8, 10% [w/v] SDS, 30% [v/v] glycerol, 5% [v/v] β-mercaptoethanol, 0.1% [w/v] bromophenol blue) and boiled for 10 min. The presence of both proteins was verified by loading 15-µL aliquots onto duplicate 13% SDS-polyacrylamide gels followed by Western blotting. Unbound FLAG-RlpA in the flow through was diluted 1:20 before loading. Purified FLAG-RlpA was also incubated with 100 µL of unbound IMAC resin and washed as above to verify the absence of non-specific binding of FLAG-RlpA to the resin itself. Pull-down assays were also performed with IMAC resin-bound His_10_-FtsK_N_ and 250 µg of purified FtsZ (purified as in ref.^66^) as a positive interaction control. All experiments were conducted in duplicate.

### Western blotting

Expression of each FtsK_N_* construct was verified by preparing whole cell lysates for SDS-PAGE and Western blotting. FtsK_N_* variants expressed in the presence and absence of *p*Bpa were diluted to an A_600_ of 0.25, equal volumes separated using a 13% SDS-polyacrylamide gel and transferred onto a nitrocellulose membrane (Bio-Rad) using a Trans-Blot® Turbo transfer system (Bio-Rad) on the Turbo Transfer setting. The blot was developed using a SNAP i.d.® 2.0 Protein Detection System (EMD Millipore) as per the manufacturer’s instructions. Primary and secondary antibodies used were mouse anti-His_6_ (Clontech) and horseradish peroxidase (HRP)-conjugated goat anti-mouse IgG (H + L) (Bio-Rad), respectively. Chemiluminescence was detected using 1 mL of HRP Detection Buffer (100 mM Tris-HCl pH 8.8, 1.25 mM luminol, 2 mM 4-iodophenylboronic acid, 5.3 mM hydrogen peroxide)^67^ with long exposure (5 min) in a Bio-Rad Gel Doc™ XR imaging system. Full-length western blots are shown in Supplementary Fig. S3.

To verify the presence of FtsK_N_, RlpA, and FtsZ in the pull-down assays, samples were separated using 13% SDS-polyacrylamide gels and transferred onto nitrocellulose membranes that were then blocked in 5% (w/v) skim milk in Tris-buffered saline (TBS) at 4 °C overnight. Mouse anti-His_6_ (Clontech) and rabbit anti-FtsZ (Cedarlane) primary antibodies were used for the detection of FtsK_N_ and FtsZ, respectively, in conjunction with the secondary antibodies alkaline phosphatase-conjugated goat anti-mouse IgG (H + L) (Sigma) and alkaline phosphatase-conjugated goat anti-rabbit IgG (H + L) (Sigma). FtsK_N_ and FtsZ blots were developed using a solution of 3.3 mg of nitro blue tetrazolium and 1.7 mg of 5-bromo-4-chloro-3-indolyl phosphate in 10 mL of alkaline phosphatase substrate buffer (100 mM Tris-HCl pH 9.5, 100 mM NaCl, 5 mM MgCl_2_). The full-length FtsZ western blot is shown in Supplementary Fig. S4. RlpA was probed directly using an HRP-conjugated mouse anti-FLAG^®^ (Sigma-Aldrich) antibody and chemiluminescence was detected using 1 mL of HRP Detection Buffer in a Bio-Rad Gel Doc^™^ XR imaging system.

### Lambda red deletion of *rlpA*

Deletion of *rlpA* from *E*. *coli* LP11-1 and W3110 was achieved using the Lambda red recombinase method described by Datsenko and Wanner^68^. Briefly, a PCR fragment containing a kanamycin resistance cassette flanked by 50 bp homology extensions of the upstream and downstream regions of *rlpA* was generated from template pKD4 using primers *rlpA*_P1 (5′-AATCCACACCCACAGGAAAATGTTGTCGAAAAGCGTGTAAGAGGTGCGCAGTGTAGGCTGGAGCTGCTTC-3′) and *rlpA*_P2 (5′-TTTGTTAACGTCATTTACAGAAATTGACACATCAGATGCCTGCTTTACGATGAATATCCTCCTTAG-3′). An overnight culture of *E*. *coli* LP11-1 or W3110 carrying the Lambda red helper plasmid pKD46 was diluted 1:100 into 20 mL Complementation Media supplemented with 150 µg/mL ampicillin and 10mM l-arabinose and grown for 2 h at 30 °C. Cells were made electrocompetent by washing three times in ice-cold filtered Milli-Q H_2_O and suspended in 200 µL ice-cold filtered Milli-Q H_2_O (100-fold concentration). Fifty-microlitres of competent cells and 100 ng of PCR product were added to a 1 mm gap electroporation cuvette and incubated on ice for 5 min. Electroporation was done using a Gene Pulser Xcell™ electroporation system (Bio-Rad) using the following parameters: 1800 V, 25 µF, 200 Ω. Shocked cells were recovered for 1 h at 30 °C in 1 mL Super Optimal Broth with catabolite repression (SOC) medium (2% [w/v] tryptone, 0.5% [w/v] yeast extract, 8.56 mM NaCl, 2.5 mM KCl, 10 mM MgCl_2_, 20 mM glucose [Fisher]), then allowed to stand overnight at room temperature before spreading onto LB agar containing 25 µg/mL kanamycin to select for kanamycin resistant transformants. Deletion of *rlpA* and insertion of the kanamycin resistance cassette in each strain was verified by colony PCR using primers homologous to a region 500 bp upstream of *rlpA* (Δ*rlpA*For; 5′-CCAGCGCGTAATGATGCTCCTGGAC-3′) and within the kanamycin resistance cassette (k1; 5′-CAGTCATAGCCGAATAGCCT-3′)^68^, as well as primers homologous to regions 50 bp upstream (Δ*rlpA*Up; 5′-CAATCCACACCCACAGGAAAATGTTCGTC-3′) and 50 bp downstream of *rlpA* (Δ*rlpA*Down; 5′-ACTTTGTTAACGTCATTTACAGAAATTGACACA-3′). PCR verification of *rlpA* deletion in each strain was completed in duplicate along with control colonies of WT *E*. *coli* W3110 and LP11-1.

## Electronic supplementary material

Supplementary Information

## References

[1] Silhavy TJ, Kahne D, Walker S (2010). The bacterial cell envelope. Cold Spring Harb. Perspect. Biol..

[2] Typas A, Banzhaf M, Gross CA, Vollmer W (2012). From the regulation of peptidoglycan synthesis to bacterial growth and morphology. Nat. Rev. Microbiol..

[3] Szwedziak P, Löwe J (2013). Do the divisome and elongasome share a common evolutionary past?. Curr. Opin. Microbiol..

[4] Goehring NW, Beckwith J (2005). Diverse paths to midcell: Assembly of the bacterial cell division machinery. Curr. Biol..

[5] Haeusser DP, Margolin W (2016). Splitsville: Structural and functional insights into the dynamic bacterial Z ring. Nat. Rev. Microbiol..

[6] Randich AM, Brun YV (2015). Molecular mechanisms for the evolution of bacterial morphologies and growth modes. Front. Microbiol..

[7] Errington J (2015). Bacterial morphogenesis and the enigmatic MreB helix. Nat. Rev. Microbiol..

[8] van den Ent F, Amos LA, Löwe J (2001). Prokaryotic origin of the actin cytoskeleton. Nature.

[9] Young KD (2010). Bacterial shape: Two-dimensional questions and possibilities. Annu. Rev. Microbiol..

[10] Michie KA, Löwe J (2006). Dynamic filaments of the bacterial cytoskeleton. Annu. Rev. Biochem..

[11] Bisson-Filho AW (2017). Treadmilling by FtsZ filaments drives peptidoglycan synthesis and bacterial cell division. Science.

[12] Mukherjee A, Dai K, Lutkenhaus J (1993). *Escherichia coli* cell division protein FtsZ is a guanine nucleotide binding protein. Proc. Natl. Acad. Sci. USA.

[13] Bi E, Lutkenhaus J (1991). FtsZ ring structure associated with division in *Escherichia coli*. Nature.

[14] Rothfield L (2003). New insights into the developmental history of the bacterial cell division site. J. Bacteriol..

[15] Strauss MP (2012). 3D-SIM super resolution microscopy reveals a bead-like arrangement for FtsZ and the division machinery: Implications for triggering cytokinesis. PLoS Biol..

[16] Wientjes FB, Nanninga N (1989). Rate and topography of peptidoglycan synthesis during cell division in *Escherichia coli*: Concept of a leading edge. J. Bacteriol..

[17] Pogliano J, Pogliano K, Weiss DS, Losick R, Beckwith J (1997). Inactivation of FtsI inhibits constriction of the FtsZ cytokinetic ring and delays the assembly of FtsZ rings at potential division sites. Proc. Natl. Acad. Sci. USA.

[18] Nanninga N (1991). Cell division and peptidoglycan assembly in *Escherichia coli*. Mol. Microbiol..

[19] van der Ploeg R (2013). Colocalization and interaction between elongasome and divisome during a preparative cell division phase in *Escherichia coli*. Mol. Microbiol..

[20] Begg KJ, Dewar SJ, Donachie WD (1995). A new *Escherichia coli* cell division gene, *ftsK*. J. Bacteriol..

[21] Liu G, Draper GC, Donachie WD (1998). FtsK is a bifunctional protein involved in cell division and chromosome localization in *Escherichia coli*. Mol. Microbiol..

[22] Barre F-X (2007). FtsK and SpoIIIE: The tale of the conserved tails. Mol. Microbiol..

[23] Demarre, G., Galli, E. & Barre, F.-X. In *DNA* Hel*icases and D*NA M*otor Proteins* (ed. Spies, M.) **973**, 245–262 (Springer New York, 2013).

[24] Draper GC, McLennan N, Begg K, Masters M, Donachie WD (1998). Only the N-terminal domain of FtsK functions in cell division. J. Bacteriol..

[25] Berezuk AM, Goodyear M, Khursigara CM (2014). Site-directed fluorescence labeling reveals a revised N-terminal membrane topology and functional periplasmic residues in the *Escherichia coli* cell division protein FtsK. J. Biol. Chem..

[26] Bigot S, Sivanathan V, Possoz C, Barre F-X, Cornet F (2007). FtsK, a literate chromosome segregation machine. Mol. Microbiol..

[27] Dubarry N, Possoz C, Barre F-X (2010). Multiple regions along the *Escherichia coli* FtsK protein are implicated in cell division. Mol. Microbiol..

[28] Dubarry N, Barre F-X (2010). Fully efficient chromosome dimer resolution in *Escherichia coli* cells lacking the integral membrane domain of FtsK. EMBO J..

[29] Grainge I (2010). FtsK - a bacterial cell division checkpoint?. Mol. Microbiol..

[30] Lesterlin C, Pages C, Dubarry N, Dasgupta S, Cornet F (2008). Asymmetry of chromosome replichores renders the DNA translocase activity of FtsK essential for cell division and cell shape maintenance in *Escherichia coli*. PLoS Genet..

[31] Grenga L, Luzi G, Paolozzi L, Ghelardini P (2008). The *Escherichia coli* FtsK functional domains involved in its interaction with its divisome protein partners. FEMS Microbiol. Lett..

[32] Di Lallo G, Fagioli M, Barionovi D, Ghelardini P, Paolozzi L (2003). Use of a two-hybrid assay to study the assembly of a complex multicomponent protein machinery: bacterial septosome differentiation. Microbiology.

[33] Butland G (2005). Interaction network containing conserved and essential protein complexes in *Escherichia coli*. Nature.

[34] Gerding MA (2009). Self-enhanced accumulation of FtsN at division sites and roles for other proteins with a SPOR domain (DamX, DedD, and RlpA) in Escherichia coli cell constriction. J. Bacteriol..

[35] Arends SJR (2010). Discovery and characterization of three new *Escherichia coli* septal ring proteins that contain a SPOR domain: DamX, DedD, and RlpA. J. Bacteriol..

[36] Yahashiri A, Jorgenson MA, Weiss DS (2015). Bacterial SPOR domains are recruited to septal peptidoglycan by binding to glycan strands that lack stem peptides. Proc. Natl. Acad. Sci. USA.

[37] Jorgenson MA, Chen Y, Yahashiri A, Popham DL, Weiss DS (2014). The bacterial septal ring protein RlpA is a lytic transglycosylase that contributes to rod shape and daughter cell separation in *Pseudomonas aeruginosa*. Mol. Microbiol..

[38] Mori H, Ito K (2006). Different modes of SecY-SecA interactions revealed by site-directed *in vivo* photo-cross-linking. Proc. Natl. Acad. Sci. USA.

[39] Yu D, Wowor AJ, Cole JL, Kendall DA (2013). Defining the *Escherichia coli* SecA dimer interface residues through *in vivo* site-specific photo-cross-linking. J. Bacteriol..

[40] Panahandeh, S. & Müller, M. In *Protein Secretion* (ed. Economou, A.) **619**, 217–240 (Humana Press, 2010).

[41] Nickerson NN (2014). Trapped translocation intermediates establish the route for export of capsular polysaccharides across *Escherichia coli* outer membranes. Proc. Natl. Acad. Sci. USA.

[42] van den Berg van Saparoea HB (2013). Fine-mapping the contact sites of the *Escherichia coli* cell division proteins FtsB and FtsL on the FtsQ protein. J. Biol. Chem..

[43] Chin JW, Martin AB, King DS, Wang L, Schultz PG (2002). Addition of a photocrosslinking amino acid to the genetic code of *Escherichia coli*. Proc. Natl. Acad. Sci. USA.

[44] Chin JW, Schultz PG (2002). *In vivo* photocrosslinking with unnatural amino acid mutagenesis. ChemBioChem.

[45] Dorman G, Prestwich G (1994). Benzophenone photophores in biochemistry. Biochemistry.

[46] Karimova G, Dautin N, Ladant D (2005). Interaction network among *Escherichia coli* membrane proteins involved in cell division as revealed by bacterial two-hybrid analysis. J. Bacteriol..

[47] Li GW, Burkhardt D, Gross C, Weissman JS (2014). Quantifying absolute protein synthesis rates reveals principles underlying allocation of cellular resources. Cell.

[48] Urban PL (2016). Clarifying misconceptions about mass and concentration sensitivity. J. Chem. Educ..

[49] Lundgren DH, Hwang S, Wu L, Han DK (2010). Role of spectral counting in quantitative proteomics. Expert Rev. Proteomics.

[50] Takase I (1987). Genes encoding two lipoproteins in the *leuS-dacA* region of the *Escherichia coli* chromosome. J Bacteriol.

[51] Gerding MA, Ogata Y, Pecora ND, Niki H, de Boer PAJ (2007). The trans-envelope Tol-Pal complex is part of the cell division machinery and required for proper outer-membrane invagination during cell constriction in *E*. *coli*. Mol. Microbiol..

[52] Potluri L (2010). Septal and lateral wall localization of PBP5, the major D, D-carboxypeptidase of *Escherichia coli*, requires substrate recognition and membrane attachment. Mol. Microbiol..

[53] Wang L, Lutkenhaus J (1998). FtsK is an essential cell division protein that is localized to the septum and induced as part of the SOS response. Mol. Microbiol..

[54] Pichoff S, Du S, Lutkenhaus J (2015). The bypass of ZipA by overexpression of FtsN requires a previously unknown conserved FtsN motif essential for FtsA-FtsN interaction supporting a model in which FtsA monomers recruit late cell division proteins to the Z ring. Mol. Microbiol..

[55] Weiss DS (2015). Last but not least: New insights into how FtsN triggers constriction during *Escherichia coli* cell division. Mol. Microbiol..

[56] Liu B, Persons L, Lee L, de Boer PAJ (2015). Roles for both FtsA and the FtsBLQ subcomplex in FtsN-stimulated cell constriction in *Escherichia coli*. Mol. Microbiol..

[57] Lopez-Garrido J, Casadesus J (2010). The DamX protein of *Escherichia coli* and *Salmonella enterica*. Gut Microbes.

[58] Born P, Breukink E, Vollmer W (2006). *In vitro* synthesis of cross-linked murein and its attachment to sacculi by PBP1A from *Escherichia coli*. J. Biol. Chem..

[59] Zou Y, Li Y, Dillon J-AR (2017). The distinctive cell division interactome of *Neisseria gonorrhoeae*. BMC Microbiol..

[60] von Rechenberg M, Ursinus A, Höltje J-V (1996). Affinity chromatography as a means to study multienzyme complexes involved in murein synthesis. Microb. Drug Resist..

[61] Vollmer W, von Rechenberg M, Höltje JV (1999). Demonstration of molecular interactions between the murein polymerase PBP1B, the lytic transglycosylase MltA, and the scaffolding protein MipA of *Escherichia coli*. J. Biol. Chem..

[62] Park AJ (2014). A temporal examination of the planktonic and biofilm proteome of whole cell *Pseudomonas aeruginosa* PAO1 using quantitative mass spectrometry. Mol. Cell. Proteomics.

[63] Keller A, Nesvizhskii AI, Kolker E, Aebersold R (2002). Empirical statistical model to estimate the accuracy of peptide identifications made by MS/MS and database search. Anal. Chem..

[64] Nesvizhskii AI, Keller A, Kolker E, Aebersold R (2003). A statistical model for identifying proteins by tandem mass spectrometry. Anal. Chem..

[65] Szklarczyk D (2015). STRING v10: protein – protein interaction networks, integrated over the tree of life. Nucleic Acids Res..

[66] Roach EJ, Kimber MS, Khursigara CM (2014). Crystal structure and site-directed mutational analysis reveals key residues involved in *Escherichia coli* ZapA function. J. Biol. Chem..

[67] Haan C, Behrmann I (2007). A cost effective non-commercial ECL-solution for Western blot detections yielding strong signals and low background. J. Immunol. Methods.

[68] Datsenko KA, Wanner BL (2000). One-step inactivation of chromosomal genes in *Escherichia coli* K-12 using PCR products. Proc. Natl. Acad. Sci. USA.

[69] Potluri L-P, Kannan S, Young KD (2012). ZipA is required for FtsZ-dependent preseptal peptidoglycan synthesis prior to invagination during cell division. J. Bacteriol..

