# Supplementary material for: Outer membrane lipoprotein RlpA is a novel periplasmic interaction partner of the cell division protein FtsK in Escherichia coli
Source: Sci Rep. 2018 Aug 28;8:12933. doi: 10.1038/s41598-018-30979-5 (PMC6113214; doi:10.1038/s41598-018-30979-5)
Supplement: Supplementary file 1 — Supplementary Information [file 41598_2018_30979_MOESM1_ESM.pdf]

Supplementary Information

Outer membrane lipoprotein RlpA is a novel periplasmic interaction partner of the cell division protein FtsK in *Escherichia coli*

Alison M. Berezuk[1], Sabrina Glavota[1], Elyse J. Roach[1], Mara C. Goodyear[1], Jonathan R. Krieger[2], and Cezar M. Khursigara[1]*

[1]Department of Molecular and Cellular Biology, University of Guelph, Guelph ON N1G 2W1;
[2]SPARC BioCentre, The Hospital for Sick Children, Toronto ON M5G 0A4

Contents:

Table S1. Protein groups detected by mass spectrometry of potential $FtsK_N$ interaction partners
Figure S1. Location of incorporated *p*-benzoyl-L-phenylalanine (*p*Bpa) residues
Figure S2. PCR verification of *rlpA* deletion
Figure S3. Complete western blots for analysis of full-length $FtsK_N$ expression
Figure S4. Complete western blot for *in vivo* analysis of $FtsK_N$ – FtsZ pull-down assay

Table S1. Protein groups detected by mass spectrometry of potential $FtsK_N$ interaction partners. Listed proteins were exclusively detected in at least one UV treated sample (absent in untreated and WT controls) of $FtsK_N$* variants W51*, D135*, D136*, Y139* and L158*. Proteins which yielded a minimum of 2 unique peptides at >95% probability in all biological replicates are listed.

The abbreviations used are as follows: GO, gene ontology; IM, inner membrane; OM, outer membrane; PG, peptidoglycan; LPS, lipopolysaccharide.

Protein	Average Spectrum Count[a]	Total Unique Peptides[b]	GO Biological Process[c]	Sample
RlpA	9	22	Cell wall organization	W51, D135, D136, Y139
YbaY	4	12	Unknown	W51, D135, D136, Y139
YbhC	26	55	Cell wall modification	W51, D136, Y139, L158
BamC	15	31	OM assembly	W51, D136, Y139
YceI	6	12	Unknown	W51, D136, Y139
MltB	10	22	Cell wall organization; PG catabolism	D135, D136, Y139
OpgG	8	14	β-glucan biosynthesis; osmotic stress response	W51, D136, L158
Pbp1A (MrcA)	10	15	Cell wall organization; PG catabolic process	W51, D136
Skp	5	4	OM protein folding	W51, L158
TolB	10	16	Bacteriocin transport	D136, Y139
YiaD	10	18	Unknown	D136, Y139
YgiM	8	8	Unknown	D136, Y139
YcjN	5	10	Transport	D136, Y139
SurA	4	8	OM protein folding	D136, Y139
GlnH	5	6	Amino acid transport	D136, Y139
MltA	4	7	Cell wall organization; PG catabolism	D136, Y139
YhdP	4	7	Unknown	D136, Y139
AmpH	3	5	Cell wall organization; PG catabolism	D136, Y139
OsmE	4	4	Osmotic stress response	D136, Y139
YdgH	3	6	Unknown	D136, Y139
PliG	2	4	Lysozyme tolerance[d]	D136, Y139
LpxL	4	2	LPS biosynthesis	W51
ArnT	3	2	LPS biosynthesis	W51
OpgH	2	2	β-glucan biosynthesis; osmotic stress response	W51
GntT	2	2	D-gluconate catabolism	W51
CyoA	13	9	Aerobic respiration	D136
BamA	11	9	OM protein assembly; cell adhesion	D136
YhiI	12	6	Protein secretion	D136
AroF	8	6	Aromatic amino acid biosynthesis	D136
MotB	6	5	Cell motility	D136
LptE	4	4	LPS transport	D136

AcrB	4	4	Drug transport	D136
NlpE	4	4	Cell adhesion	D136
LolA	4	4	Lipoprotein transport	D136
YidC	3	3	IM protein assembly	D136
BamB	3	3	OM protein assembly	D136
OmpT	3	3	Proteolysis	D136
FlgH	3	3	Cell motility	D136
MCP2 (Tar)	3	3	Chemotaxis	D136
FlgK	2	2	Cell motility	D136
Slp	4	2	OM stabilization[d]	D136
LamB	2	2	Maltodextrin transport	D136
DacA	3	2	Cell wall organization; PG biosynthesis	D136
DadA	2	2	D-alanine catabolism	D136
DamX	2	2	Cell division	D136
FlgI	2	2	Cell motility	D136
BcsC	2	2	Cellulose biosynthesis	D136
BorD	2	2	Response to Mg^{2+}	D136
Slt	2	2	Cell wall organization; PG catabolism	D136
Blc	2	2	DNA damage response	D136
DacC	2	2	Cell wall organization; PG biosynthesis	D136
EcoT	2	2	Protease inhibition[d]	D136
DppA	11	10	Protein transport; peptide chemotaxis	Y139
RbsB	3	3	D-ribose transport; chemotaxis	Y139
PtnD	3	2	Mannose transport	Y139
YeaY	2	2	Unknown	Y139
SfgH1 (FrmB)	2	2	Formaldehyde metabolism	Y139
YddW	2	2	Unknown	Y139
YraP	2	2	Unknown	Y139
FtsN	2	2	Cell division	Y139
MCP4 (Tap)	2	2	Chemotaxis	Y139
TamB	2	2	Protein secretion	Y139
YdbH	2	2	Unknown	Y139

[a] Calculated using the spectrum count detected across all samples.
[b] Sum of all unique peptides detected across all samples.
[c] Gene ontology biological process, as reported in the UniProtKB *E. coli* K12 database.
[d] GO biological process annotation not provided, term determined based on molecular function.

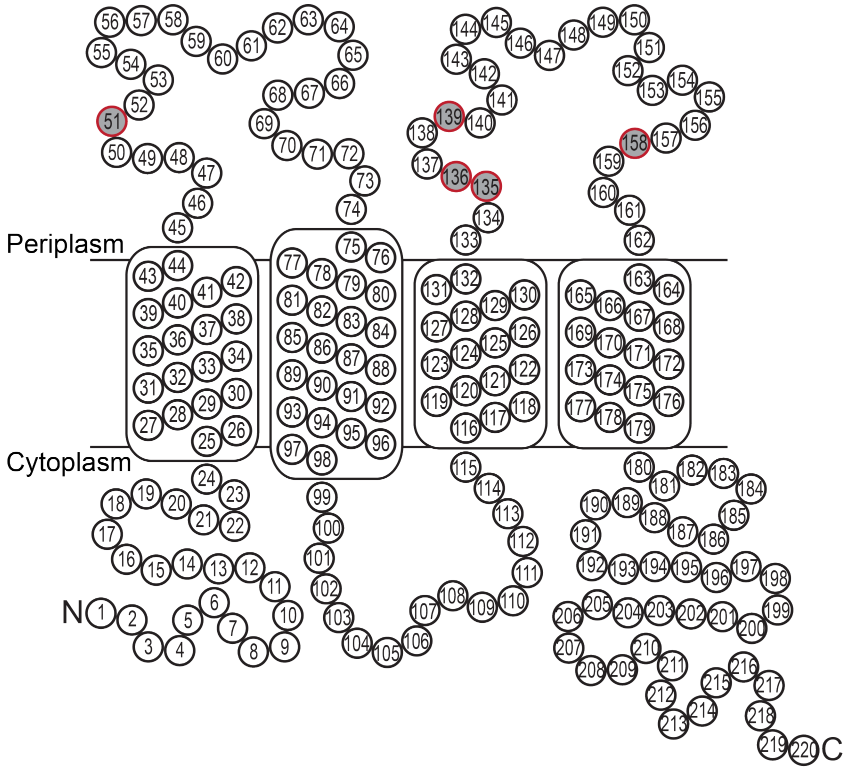

Figure S1. Location of incorporated *p*-benzoyl-L-phenylalanine (*p*Bpa) residues. Membrane topology of $FtsK_N$ illustrating the position of each amino acid targeted for replacement by *p*Bpa (*shaded red circles*) to produce single photo-modified protein variants ($FtsK_N$*).

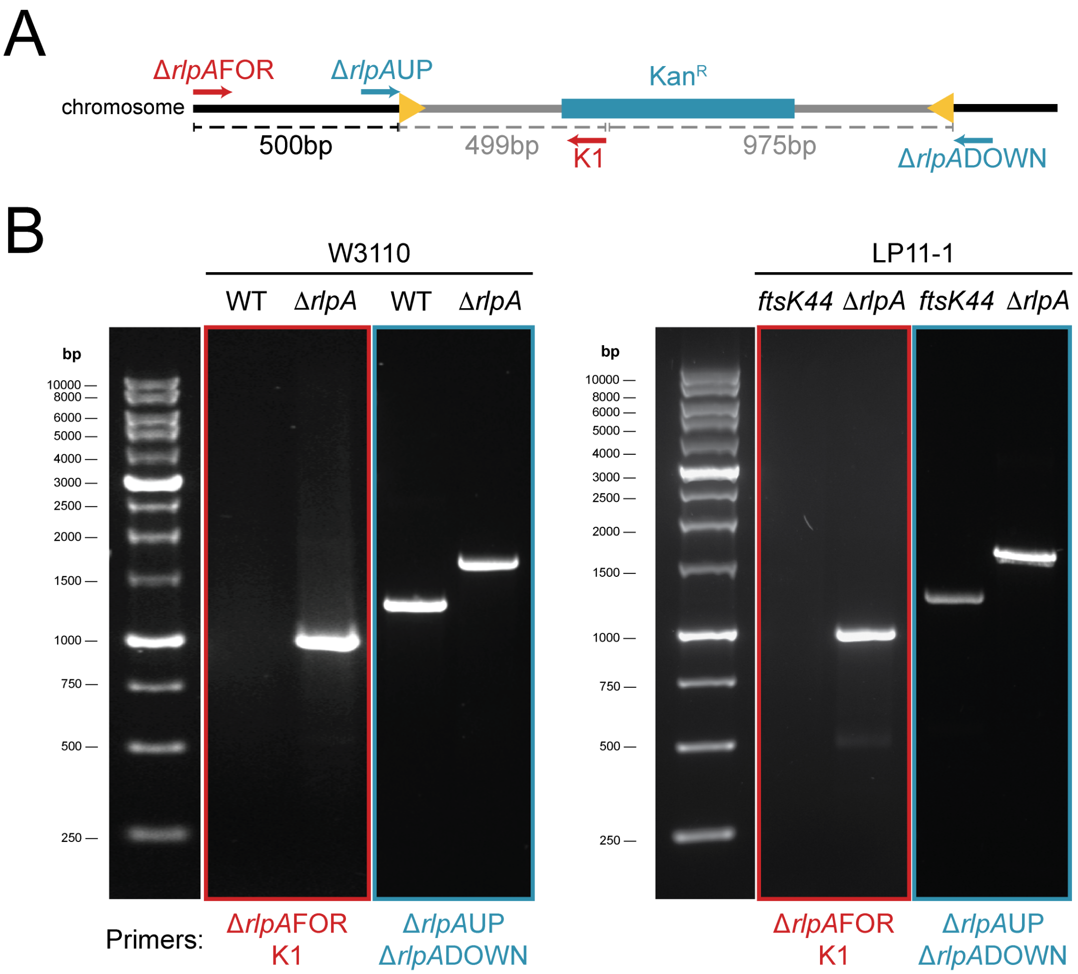

Figure S2. PCR verification of *rlpA* deletion. *A*, genomic arrangement, and *B*, colony PCR of the *rlpA* deletions to verify insertion of the kanamycin resistance cassette (Kan^R). Boundaries of the Kan^R insert are denoted by the yellow triangles and grey line. Red and blue arrows indicate binding positions of the primers used to verify insertion of Kan^R. Δ*rlpA*FOR binds 500 bp upstream of *rlpA* and K1 binds 499 bp downstream of the 5' boundary of the Kan^R insert, resulting in a PCR amplicon of 999 bp. Δ*rlpA*UP and Δ*rlpA*DOWN bind 50 bp upstream and downstream of *rlpA*, respectively, resulting in a PCR amplicon of 1189 bp in the parental strains (noted above each gel) and 1574 bp in the deletion strains. PCR verification of *rlpA* deletion was completed in duplicate along with colonies of WT *E. coli* W3110 and LP11-1 as negative controls.

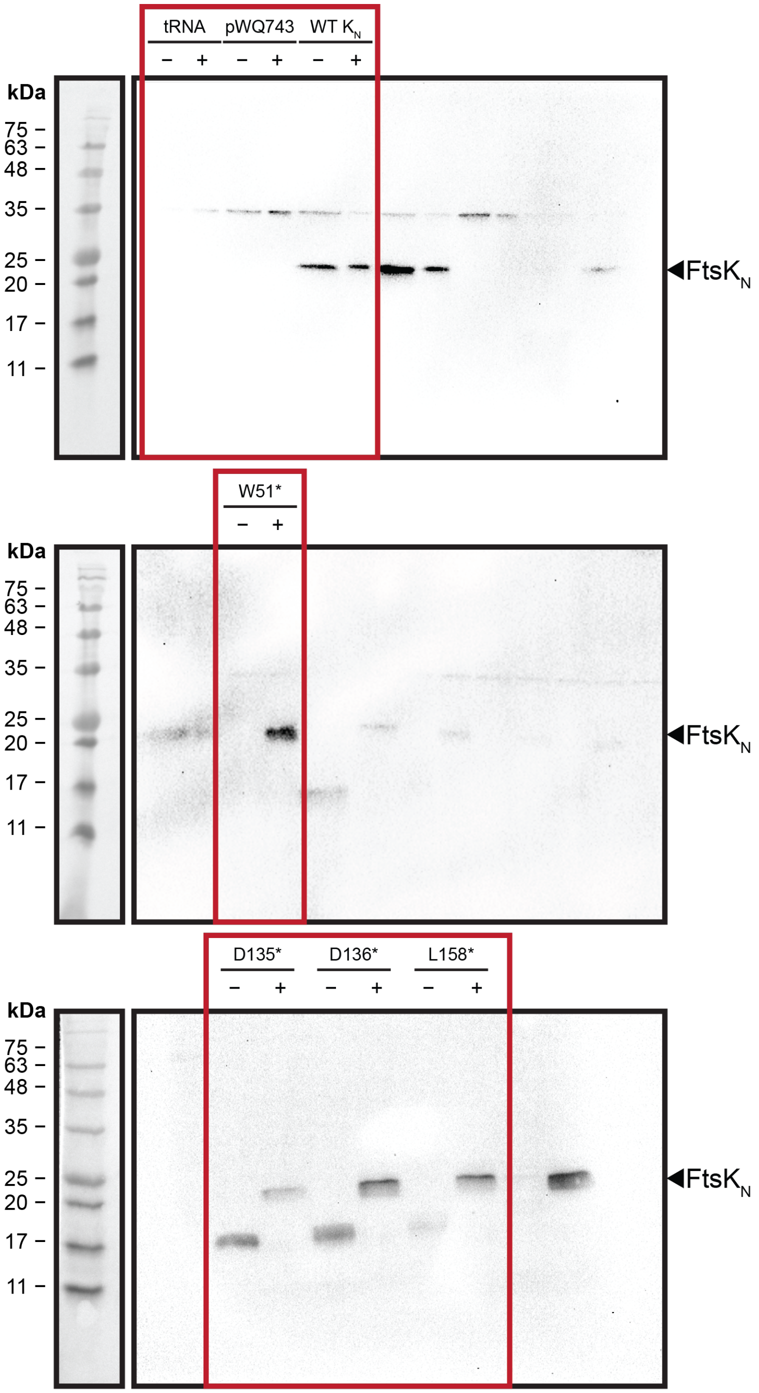

Figure S3. Complete western blots for analysis of full-length FtsK$_N$ expression. All western blots of FtsK$_N$ amber mutant expression from *Figure 1* were probed using a mouse anti-His$_6$ primary antibody and a horseradish peroxidase-conjugated goat anti-mouse secondary antibody. tRNA and pWQ743 represent control cells harbouring the mutant tRNA/tRNA synthease and empty vector, respectively. The lanes shown in *Figure 1* are highlighted by the red boxes.

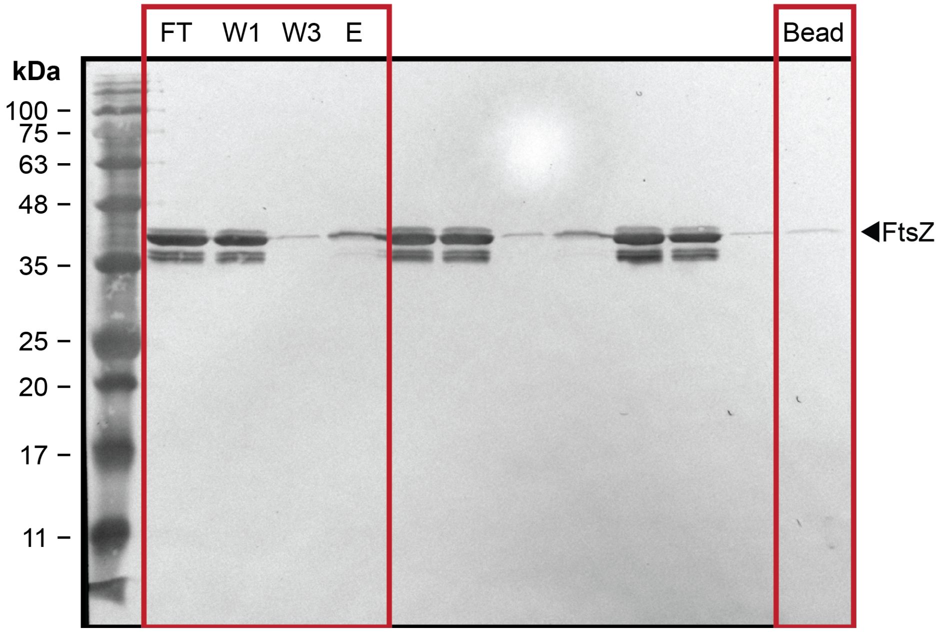

Figure S4. Complete western blot for *in vitro* analysis of FtsK$_N$ – FtsZ pull-down assay. Western blot of pull-down assay between FtsZ (prey) and His$_{10}$-FtsK$_N$ (bait) from *Figure 4* was probed using a mouse anti-FtsZ primary antibody and an alkaline phosphatase-conjugated goat anti-mouse secondary antibody. *'FT'* – flow through; *'W1, W3'* – wash fractions 1, and 3; *'E'* – elution with 1 M imidazole; *'Bead'* – elution fraction of FtsZ incubated with empty IMAC resin (negative control). The lanes shown in *Figure 4* are highlighted by the red boxes.
